# The Effect of Catheter Ablation on Left Atrial Size and Function for Patients with Atrial Fibrillation: An Updated Meta-Analysis

**DOI:** 10.1371/journal.pone.0129274

**Published:** 2015-07-06

**Authors:** Bin Xiong, Dan Li, Jianling Wang, Laxman Gyawali, Jinjin Jing, Li Su

**Affiliations:** Department of Cardiology, The Second Affiliated Hospital of Chongqing Medical University, The Chongqing Cardiac Arrhythmias Service Center, Chongqing, China; University of Minnesota, UNITED STATES

## Abstract

**Background:**

Catheter ablation (CA) for atrial fibrillation (AF) is now an important therapeutic modality for patients with AF. However, data regarding changes in left atrial (LA) function after CA have indicated conflicting results depending on the AF types, follow-up period, and the analytical imaging tools. The objective of this review was to analyze the effect of CA on the LA size and function for patients with AF.

**Methods:**

We searched for studies regarding LA size and function pre- and post-ablation in PubMed, Embase, the Cochrane Library, and Web of Knowledge through May 2014. LA function was measured by LA ejective fraction (LAEF), LA active ejective fraction (LAAEF), or both. Total and subgroup analyses were implemented using Cochrane Review Manager Version 5.2. Weighted mean differences with 95% confidence intervals were used to express the results of continuous outcomes using fixed or random effect models. I^2^ was used to calculate heterogeneity. To assess publication bias, Egger’s test and Begg’s funnel plot were performed using Stata 12.0.

**Results:**

Twenty-five studies (2040 enrolled patients) were selected for this meta-analysis. The LA diameter (LAD), maximum LA volume, and minimal LA volume were significantly decreased post-ablation, as compared with those at a pre-ablation visit. Compared with the pre-ablation outcomes, we found no significant differences in LAEF/LAAEF at a post-ablation follow-up. Decreases in LA volume and LAEF remained significant post-ablation for paroxysmal AF (PAF); however, the LAEF was insignificant changes in persistent AF (PeAF). Heterogeneity was significant in spite which individual study was excluded. A publication bias was not found. In a meta-regression analysis, we did not find any factor that contributed to the heterogeneity.

**Conclusion:**

With CA, LA volumes and LAD were decreased significantly in patients with AF; LAEF was not significant changes in patients with PeAF but decreased in those with PAF.

## Introduction

Atrial fibrillation (AF) is the most commonly sustained tachyarrhythmia in clinical practice. It is associated with an increase in disease-related hospitalizations; a reduction in quality of life; complications such as congestive heart failure (HF), thromboembolism, and stroke; and an increased mortality risk [1–4]. Catheter ablation (CA) is considered an efficient mainstream therapy and potentially curative treatment for drug-refractory symptomatic AF [5, 6]. After successful ablation, patients with AF would experience improved left atrial (LA) function because of a reduction in AF burden. Nevertheless, it is noted that extensive atrial scar tissue formation produced by CA may result in adverse reactions in atrial function in AF patients. Although Jeevanantham et al. [7] reported successful CA for AF patients does not appear to adversely impact LA function, recent studies that investigated the impact of CA on LA function reported inconsistent results. Therefore, the purpose of this study was to update evidence regarding the effect of CA on the LA size and function in patients with AF.

## Methods

### Search Strategy

We performed a search for articles pertaining to CA in AF patients using the key words “atrial fibrillation,” “catheter ablation,” “atrial size,” “left atrial function,” and “left atrium function.” We searched for all relevant studies, without any language limitations, in PubMed, Embase, the Cochrane Library, and Web of Knowledge through May 2014. Manual searches were also performed of the bibliographies.

### Inclusion and Exclusion Criteria

The inclusion criteria were as follows: (1) randomized control trials (RCTs) or nonrandomized control trials were included; (2) follow-up imaging was performed no less than 3 months post-ablation; (3) primary outcome measurements changed regarding maximum LA volume (LAVmax), minimum LA volume (LAVmin), LA diameter (LAD), LA ejection fraction (LAEF; LAEF = [LAVmax − LAVmin]/LAVmax), LA active emptying fraction (LAAEF; LAAEF = {LA mid-diastolic volume just before atrial contraction [LAVmid]–LAVmin}/LAVmid), A wave velocity (A; *defined as the peak velocities of late transmitral flow measured by pulsed-wave Doppler echocardiography [DE]*), and the A' wave velocity (A'; *defined as the velocities of the mitral annulus during atrial contraction as measured by pulsed-wave tissue Doppler echocardiography [TDE]*) [8].

Exclusion criteria were as follows: (1) surgical ablation; (2) left ventricular ejection fraction (LVEF) of <50% or included HF patients in each enrolled study; (3) significant valvular disease including a stenotic valvular lesion or moderate-to-heavy regurgitation after valvular replacement; (4) heart dysfunction was caused by structural heart disease or another disease; (5) the LA parameters, as detailed previously, were not reported either pre- or post-ablation; and (6) median and inter-quartile range outcomes were reported.

### Data Extraction and Quality Evaluation

Two reviewers (Xiong and Li) assessed the quality of each study and then independently extracted data from the included studies; another author (Wang) checked the data. The extracted information were: (1) basic information regarding those studies, including country and publication year; (2) the number of patients in the study; (3) patient characteristics; (4) type of catheter ablation performed for the treatment group; and (5) outcome measures, as previously defined. Any disagreement was resolved by discussion with a third party (Wang).

To evaluate the quality of the included studies, the following aspects had been performed, including (1) research design; (2) the representativeness of the enrolled patients; (3) the bias of loss to follow-up; and (4) other biases and limitations.

### Statistical Analysis

Cochrane Review Manager Version 5.2 and Stata 12.0 were used to perform the statistical analysis. Weighted mean differences (WMDs) with 95% confidence intervals (CIs) were used for expressing continuous outcomes. Statistical heterogeneity was tested using the χ^2^ test and was quantified using the I^2^ statistic; significant heterogeneity was defined as a *P* of <0.10 or an I^2^ of >50%. Data were pooled using a fixed effect or random effect model, based on whether the absence of significant heterogeneity existed. If the absence of heterogeneity was significant, the fixed effect model was performed, but if not, the random effect model was performed. Publication bias was evaluated using Egger’s test and Begg’s funnel plot with Stata 12.0; statistical significance was defined as a *P* of <0.05.

## Results

### Study Characteristics

**Fig 1:**
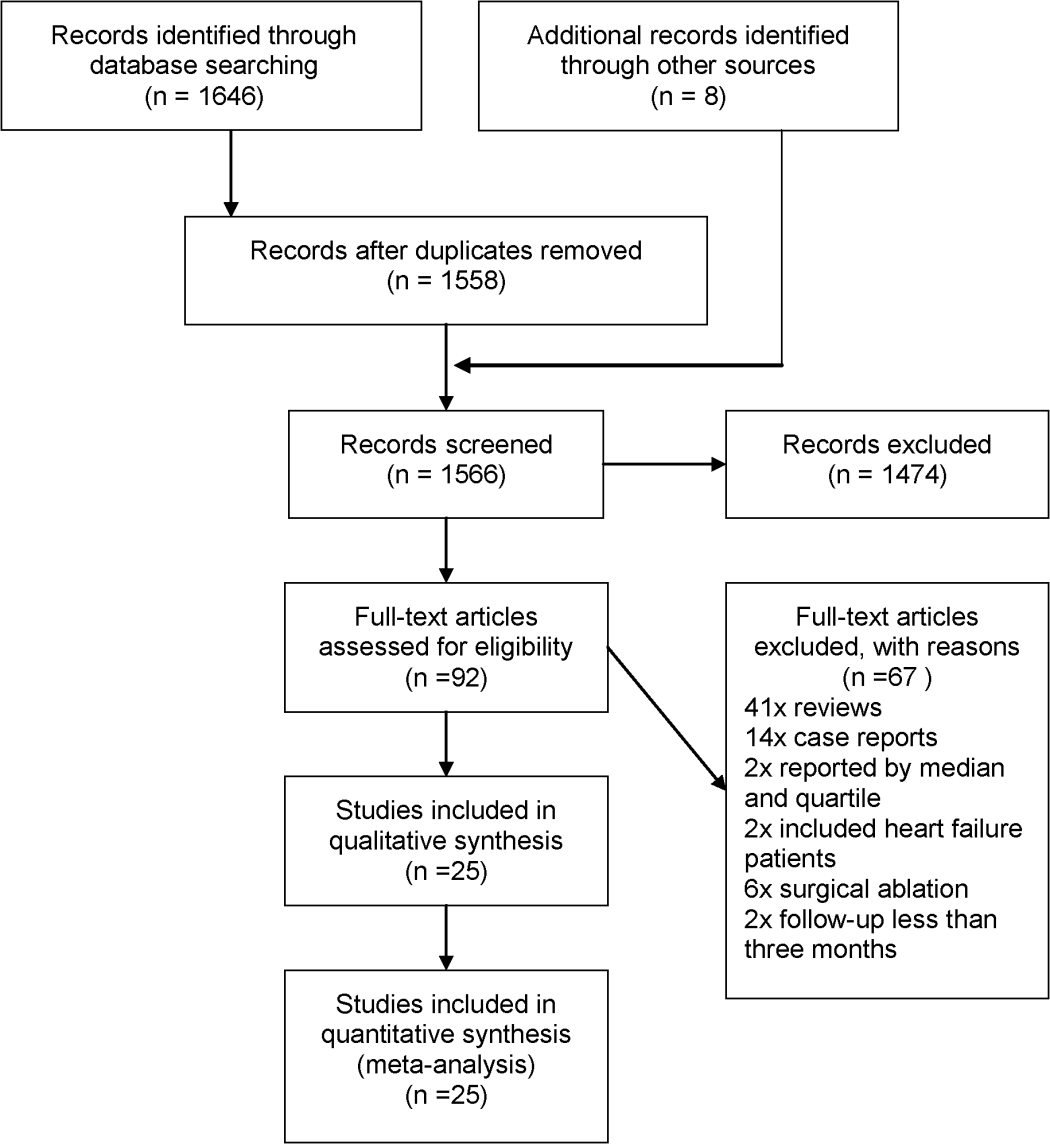
Flow chart of the literature search and study selection processes.

We identified 1566 references from electronic databases using the previously described strategy. According to the inclusion criteria, 92 citations were retrieved and required further evaluation after screening the title, abstract, or both. Forty-one reviews and 14 case reports were excluded. Two studies reported median and inter-quartile range outcomes; 6 studies included surgical ablation; 2 studies had a follow-up of <3 months; and 2 studies included HF patients. Finally, 25 studies (2040 enrolled patients) were selected for this meta-analysis [9–33]. The selection process is demonstrated in a flow chart (Fig 1). The characteristics of each included study are listed in Table 1. The primary results of each included study are shown in Table 2.

**Table 1:** Characteristics of the studies included in the current review.

Study	Area	Number of Patients	Age (yrs)	Men	Paroxysmal AF Persistent AF Permanent AF	Follow-Up (mon)	Duration of AF	Comorbidities	Medications	Type of Ablation	Type of Imaging	LVEF (%)	Patients with Recurrence	Success Rate
Dagres [9]	Greece	289	56±9	214	289	12	68±58mon	HTN (43%);	NA	PVCA	TTE	62±10	123	166/289
2009				(74%)	NA			DM (5%);						(57%)
					NA			CAD (5%)						
Erdei [10]	Hungary	36	57±9	26	36	12	6.7±7.3yrs	HTN (75%);	AAD	CCA	TTE	63±5	21	15/36
2012				(72%)	NA			IHD (11%);						(42%)
					NA			OB (28%)						
Hof [11]	Netherlands	206	57±10	165	114	16	7±6yrs	HTN (35%);	AAD	PVAI	CMR	NA	37	169/206
2013				(80%)	92			IHD (10%)	(Class I and III)					(82%)
					NA									
Jahnke [12]	Germany	41	57±10	28	25	12	NA	HTN (66%);	NA	PVI	CMR	58±5	10	31/41
2011				(68%)	16			DM (5%);						(76%)
					NA			CAD (20%);						
								HLP (59%)						
Machino-	Japan	123	60±9	104	NA	18	5.2±4.3yrs	HTN (56%);	AAD	PVI	TTE	NA	45	78/123
Ohtsuka[13]				(85%)	123			DM (7%);						(63%)
2013					NA			CAD (6%)						
Masuda [14]	Japan	115	62±10	82	92	3	44.6±51.9mon	HTN (50%);	AAD	PVCA	MDCT	67±7	32	83/115
2012				(71%)	23			DM (13%)						(72%)
					NA									
Montserrat	Spain	158	53±11	120	77	6	52±34mon	HTN (44%)	AAD	RFCA	TTE	59±9	82	76/158
[15] 2011				(76%)	77									(48%)
					NA									
Nori [16]	America	29	54±11	18	16	3	4.1±3.4yrs*	HTN (48%);	NA	PVAI	CMR	63±11*	NA	NA
2009				(62%)	13		2.0±1.0yrs#	DM (17%);				60±10#		
					NA			CAD (38%);						
								HLP (55%)						
Rodrigues	Brazil	28	53±13	22	28	8	6 yrs	HTN (39%)	Amiodarone;	PVCA	TTE	NA	11	17/28
[17] 2009				(79%)	NA		(3mon-20yrs)		Propafenone;					(61%)
					NA				β-blocker					
Teh [18]	Australia	11	59±8	8	7	10	5.6±4.8yrs	NA	NA	RFCA	TTE	60±7	NA	NA
2012				(73%)	4									
					NA									
Tops [19]	Netherlands	148	54±9	117	112	13	5.3±4.5yrs	HTN (42%);	AAD;	PVI	TTE	57±7	49	99/148
2011				(79%)	36			CAD (6%)	ACEI (49%)					(67%)
					NA									
Yoshida [20]	Japan	67	64±8	58	34	6	NA	NA	NA	PVI	TTE	NA	11	56/67
2013				(87%)	33									(84%)
					NA									
Reant [24]	France	48	53±9*	40	37	11	6±5yrs*	NA	β-blocker (29%);	PVI	TTE	62±5*	13	35/48
2005			55±11&	(83%)	NA		12±9yrs&		Amiodarone(2%);			53±8&		(73%)
					11&				Flecainide (14%)					
Delgado	Spain	34	53±13	24	23	6	90±72mon	HTN (24%)	AAD	CPVA	3D-TTE	NA	13	21/34
[29] 2008				(70%)	6									(62%)
					5									
Verma [27]	America	67	56±10	49	40	6	5.8±5.1yrs	HTN (31%);	AAD	PVAI	TTE,	50±13	NA	NA
2006				(73%)	27			DM (9%);			CT			
					NA			CAD (19%);						
								VHD (15%)						
Lemola [23]	America	36	55±11	24	27	5	5±4yrs	CAD (9%)	NA	LACA	CT	56±5	NA	NA
2005				(67%)	NA									
					9&									
Perea [33]	Spain	55	52±11	44	41	12	8.4±8yrs	HTN (22%);	AAD	CPVA	CMR	60±9	17	38/55
2008				(80%)	14			SHD (16%)						(69%)
					NA									
Muller [32]	Switzerland	91	59±8	79	72	6	6.4±5.8yrs	HTN (33%);	ACEI and/or ARB(30%);	PVI	TTE	NA	21	70/91
2008				(87%)	11			IHD (7%)	Diuretic (15%);					(77%)
					8				Amiodarone (24%);					
									Sotalol (12%);					
									Ic (31%);					
									β-blocker (43%)					
Marsan [31]	Netherlands	57	56±9	44	43	8	4.6±4.1yrs	HTN (44%);	Amiodarone;	RFCA	3D-TTE	57±9	19	38/57
2008				(77%)	14			DM (11%);	Propafenone;					(67%)
					NA			CAD (5%)	Flecainide;					
									Sotalol;					
									ACEI and/or ARBs(46%)					
Beukema	Netherlands	105	53±10	88	52	15	6±5.1yrs*	HTN (26%);	AAD	PVI	TTE	54±4	34	71/105
[22] 2005				(84%)	53		7.6±6yrs#	DM (5%)						(68%)
					NA									
Choi [28]	Korea	33	56±10	27	21	3	63±47mon	HTN (21%);	ACEI or ARB (24%);	RFCA	TTE	53±6	NA	NA
2008				(82%)	12			DM (6%);	CCB (30%);					
					NA			CAD (6%)	β-blocker (15%);					
									Amiodarone (30%);					
									Propafenone (30%);					
									Flecainide (6%)					
Liu [30]	China	120	60±9	80	120	12	2.6±1.4yrs	NA	Amiodarone;	CPVA	TTE	67±3	42	78/120
2008				(83%)	NA				Losartan	SPVI				(65%)
					NA									
Tops [26]	Netherlands	57	53±8	45	35	3	6±5yrs	HTN (30%);	AAD	RFCA	TTE	55±7	18	39/57
2006				(79%)	18			CAD (7%);						(68%)
					4			VHD (11%)						
Lemola [21]	America	41	54±12	33	25	4	5±3yrs	HTN (21%);	NA	LACA	CT	55±8	8	33/41
2004				(80%)	NA			SHD (41%)						(80%)
					16									
Tsao [25]	China	45	60±13	36	45	21	NA	NA	AAD	PVI	CMR	NA	10	35/45
2005	Taiwan			(80%)	NA									(77%)
					NA									

* Paroxysmal Atrial Fibrillation

# Persistent Atrial Fibrillation

& Chronic Atrial Fibrillation

NA = Not Available; mon = months; yrs = years

HTN = Hypertension; DM = Diabetes Mellitus; CAD = Coronary Artery Disease; OB = Obesity; IHD = Ischemic Heart Disease; HLP = Hyperlipidemia; VHD = Valvular Heart Disease; SHD = Structural heart disease.

ACEI = Angiotensin Converting Enzyme Inhibitors; ARB = Angiotensin Receptor Blocker; CCB = Calcium-channel Blocker; AAD = Anti-Arrhythmic Drugs.

RFCA = Radiofrequency Catheter Ablation; CCA = Cryoballoon Catheter Ablation; PVI = Pulmonary vein isolation; PVAI = Pulmonary Vein Antrum Isolation; CPVA/PVCA = Circumferential Pulmonary Vein Catheter Ablation; SPVI = Segmental Pulmonary Vein Isolation; LACA = Radiofrequency Left Atrial Circumferential Ablation. CMR = Cardiac Magnetic Resonance Imaging; TTE = Transthoracic Echocardiography; TEE = Transesophageal Echocardiography; MDCT = Multidetector Computed Tomography.

Atrial fibrillation recurrence is defined as documented by body surface 12-lead electrocardiogram (ECG) or 24-hour Holter ECG lasting 30 seconds, despite being symptomatic or not, at any time from 3 months after catheter ablation.

**Table 2:** Primary outcome variables before and after ablation.

Study		LAD	LAD	LAVmax	LAVmax	LAVmin	LAVmin	LAEF	LAEF	LAAEF	LAAEF	A Wave	A Wave	A' Wave	A' Wave
		Pre-ablation	Post-ablation	Pre-ablation	Post-ablation	Pre-ablation	Post-ablation	Pre-ablation	Post-ablation	Pre-ablation	Post-ablation	Pre-ablation	Post-ablation	Pre-ablation	Post-ablation
Dagres [9]		42±6*	41±5*	NA	NA	NA	NA	NA	NA	NA	NA	59±23*	53±12*	NA	NA
2009															
Erdei [10]		54±6(NR) *	56±5(NR) *	67±20(NR) *	69±15(NR) *	30±12(NR) *	32±11(NR) *	55±8(NR) *	55±9(NR) *	NA	NA	NA	NA	10.7±2.7(NR) *	10.8±3.1(NR) *
2012		55±5(R) *	59±6(R) *	73±23(R) *	81±24(R) *	38±19(R) *	44±20(R) *	48±11(R) *	47±11(R) *					9.8±2.1(R) *	10.2±2.7(R) *
Hof [11]		NA	NA	116.6±27.7*	104.1±25.3*	62.8±20*	57.9±18.9*	43.8±9.3	41.2±9.6	27.9±9.5	25.4±9.5	NA	NA	NA	NA
2013				135.6±35.9#	121.5±34.8#	80.2±32.1#	73.8±27.1#								
Jahnke		NA	NA	98±18(NR)	83.7±19.3(NR)	68.2±23.7(NR)	50.4±18.4(NR)	31.4±17.3(NR)	40.7±13.2(NR)	NA	NA	NA	NA	NA	NA
[12] 2011				116.7±20.3(R)	108±19.8(R)	80.2±24.9(R)	71.3±23.8(R)	31.8±15.2(R)	34.9±13.9(R)						
Machino-		NA	NA	48±25(NR) #	34±16(NR) #	40±19(NR) #	23±12(NR) #	24±17(NR) #	36±14 NR) #	NA	NA	54±12#	63±19#	NA	NA
Ohtsuka				57±23(R) #	59±22(R) #	47±16(R) #	49±20(R) #	21±16(R) #	17±14(R) #						
[13] 2013															
Masuda		NA	NA	57.3±17.7*	53.2±14.9*	NA	NA	47.4±11.8*	44.9±10.6*	NA	NA	NA	NA	NA	NA
[14] 2012				65.8±28.4#	53.4±22.8#			32.7±11.4#	39.1±11.5#						
Montserrat		42±6*	41±6*	53±16*	47±15*	30±12*	28±11*	44±16*	40±13*	37±23*	44±25*	NA	NA	NA	NA
[15] 2011		44±6#	43±5#	64±20#	56±18#	46±18#	39±16#	28±16#	31±17#						
Nori [16]		NA	NA	37±6.4*	28.5±5.9*	19.7±5.7*	16.3±5.9*	47.3±10.1*	42.7±9.4*	33.4±8.3*	26.2±7.9*	NA	NA	NA	NA
2009				41.4±8.7#	36.7±10#	31.7±9.3#	24±8.8#	24.1±11.8#	34.8±10.2#						
Rodrigues		41±7*	40±6*	56±21(NR)*	58±20(NR)*	30±15*	34±15*	47±8*	43±8*	NA	NA	55±15*	58±19*	7.9±2.3*	8.1±2.8*
[17] 2009				53±14(R)*	57±20(R)*										
Teh [18]		45±7	42±6	76±30	63±23	NA	NA	NA	NA	NA	NA	NA	NA	NA	NA
2012															
Tops [19]		43±4	40±4	31±7	21±6	19±6	12±5	41±14	46±11	NA	NA	NA	NA	NA	NA
2011															
Yoshida		38±7*	40±8*	NA	NA	NA	NA	NA	NA	NA	NA	NA	NA	NA	NA
[20] 2013		41±6#	40±8#												
Reant [24]		59.7±7.3*	53.19±7.7*	NA	NA	NA	NA	NA	NA	30.9±13.5*	34±11.3*	NA	NA	NA	NA
2005		68.4±8.1&	60.68±6.5&							5.4±3.6&	21.8±11&				
Delgado		40±6(NR)	39±6(NR)	50±11(NR)	45±10(NR)	26±13(NR)	24±8(NR)	49±19(NR)	48±18(NR)	25±21(NR)	26±21(NR)	31±27(NR)	30±26(NR)	NA	NA
[29] 2008		42±7(R)	43±9(R)	64±19(R)	53±22(R)	32±12(R)	30±15(R)	49±14(R)	43±13(R)	23±19(R)	18±12(R)	22±36(R)	42±16(R)		
Verma [27]		45.9±10.2	44.4±4.5	94.5±28.1	85.8±18.2	78.6±23.8	66.8±13.9	16.7±5.8	22.1±5.4	NA	NA	42.8±20.9	61.9±17.3	NA	NA
2006															
Lemola		NA	NA	121±40*	95±30*	87±39*	78±27*	32±13*	21±8*	NA	NA	NA	NA	NA	NA
[23] 2005															
Perea [33]		NA	NA	98±19.9(NR)	84.9±17.1(NR)	58.6±16.1(NR)	52.2±12.1(NR)	40.2±11.5(NR)	38.1±9.8(NR)	NA	NA	NA	NA	NA	NA
2008				126.2±32.8(R)	103.5±28.1(R)	78.4±22.2(R)	75.8±24.3(R)	37.4±10.1(R)	26.9±10.2(R)						
Muller [32]		56±8	53±7	59.6±21.3	51±15.5	NA	NA	NA	NA	NA	NA	59.7±20.4	59±16.1	8.9±2.9	9.8±3.4
2008															
Marsan		NA	NA	26±8(NR)	23±7(NR)	13±5(NR)	10±4(NR)	52±10(NR)	58±10(NR)	22±8(NR)	33±9(NR)	NA	NA	NA	NA
[31] 2008				31±8(R)	32±8(R)	16±7(R)	18±6(R)	47±13(R)	42±11(R)	24±7(R)	15±9(R)				
Beukema		40.5±4.4(NR) *	37.5±3.5(NR) *	NA	NA	NA	NA	NA	NA	NA	NA	NA	NA	NA	NA
[22] 2005		44±5.8(NR) #	40±4.5(NR) #												
		45±6.5(R) #	49±5.4(R) #												
Choi [28]		41±5.4	39±6.4	63.4±20.7	50.7±16.6	43.8±18.2	35.1±12.9	31.8±12.8	30.9±10	NA	NA	60.7±22.7	44.8±16.7	9.7±1.9	7.6±1.6
2008															
Liu	CPVA	33.8±3.6(NR) *	32.2±2.5(NR) *	NA	NA	NA	NA	NA	NA	NA	NA	NA	NA	NA	NA
[30]		34.9±2.8(R) *	34.1±1.9(R) *												
2008	SPVI	34.8±2.8(NR) *	35±2.4(NR) *	NA	NA	NA	NA	NA	NA	NA	NA	NA	NA	NA	NA
		35.4±2.7(R) *	38.4±2.8(R) *												
Tops [26]		45±3(NR)	42±3(NR)	59±12(NR)	50±11(NR)	37±9(NR)	31±7(NR)	NA	NA	NA	NA	NA	NA	NA	NA
2006		45±3(R)	48±3(R)	63±7(R)	68±8(R)	43±7(R)	47±7(R)								
Lemola		NA	NA	115±39(NR)	97±35(NR)	NA	NA	NA	NA	NA	NA	NA	NA	NA	NA
[21] 2004				128±80(R)	135±70(R)										
Tsao [25]		33.5±5.9(NR) *	32.5±6.9(NR) *	61.5±19.1(NR) *	56.6±17.1(NR) *	NA	NA	NA	NA	NA	NA	NA	NA	NA	NA
2005		34.1±6.6(R) *	36.2±6.4(R) *	61.1±17.5(R) *	78.7±25.3(R) *										

* Paroxysmal Atrial Fibrillation

# Persistent Atrial Fibrillation

& Chronic Atrial Fibrillation

NR = Not Recurrence; R = Recurrence; NA = Not Available

LAD = left atrial diameter; LAVmax = maximum left atrial volume; LAVmin = minimum left atrial volume; LAEF = left atrial ejective fraction; LAAEf = left atrial active ejective fraction; A wave = A wave velocity; A' wave = A' wave velocity

Other abbreviations and AF Recurrence defined as previously detailed.

All patients had underwent CA, one study [15] had repeated ablation. The majority of studies performed radiofrequency catheter ablation (RFCA), only one study [10] implemented cryoablation. Twelve studies [10, 12, 13, 17, 21, 22, 25, 26, 29–31, 33] had reported changes in LAD, LA volumes, or function on the basis of AF recurrence (AF recurrence defined as documented by body surface 12-lead electrocardiogram (ECG) or 24-hour Holter ECG lasting 30 seconds, despite being symptomatic or not, at any time from 3 months after CA [34]). Liu et al. [30] had compared two different treatment strategies [circumferential pulmonary vein ablation (CPVA) vs. segmental pulmonary vein isolation (SPVI)] on left atrial size in patients with lone paroxysmal AF (PAF). Nineteen studies [11, 12, 14–16, 18–24, 26–29, 31–33] had included patients with paroxysmal or non-paroxysmal AF, five studies [9, 10, 17, 25, 30] only included patients with PAF, one study [13] only included patients with persistent AF (PeAF), six studies [21, 23, 24, 26, 29, 32] included patients with permanent AF (only 49 enrolled patients). There were some co-morbidities including hypertension (HTN), diabetes mellitus (DM), and coronary artery disease (CAD) et al. in the majority of enrolled patients.

### Quantitative Data Synthesis

**Fig 2:**
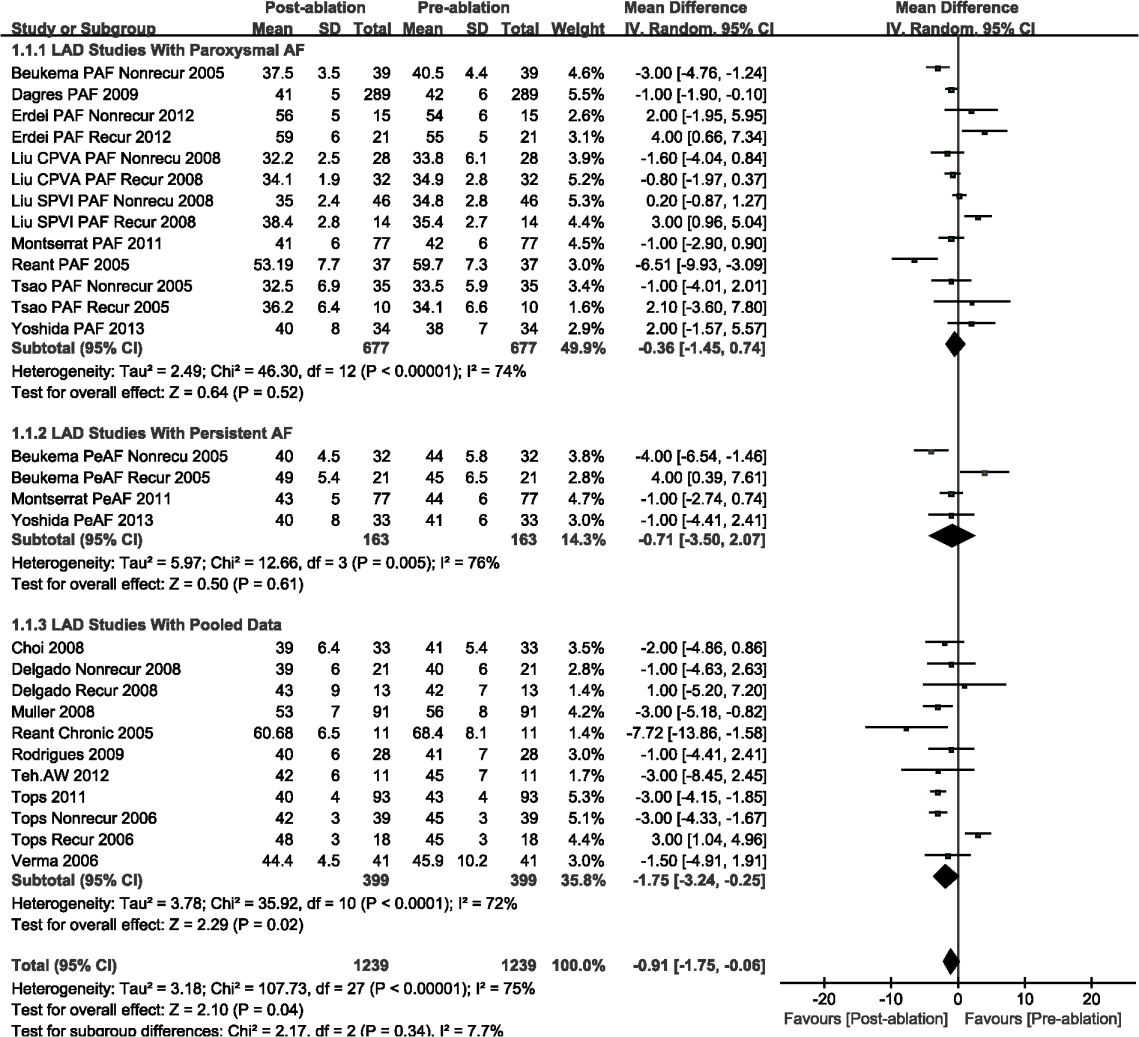
A forest plot of comparison: changes in left atrial diameter (LAD) pre-ablation and post-ablation.

**Fig 3:**
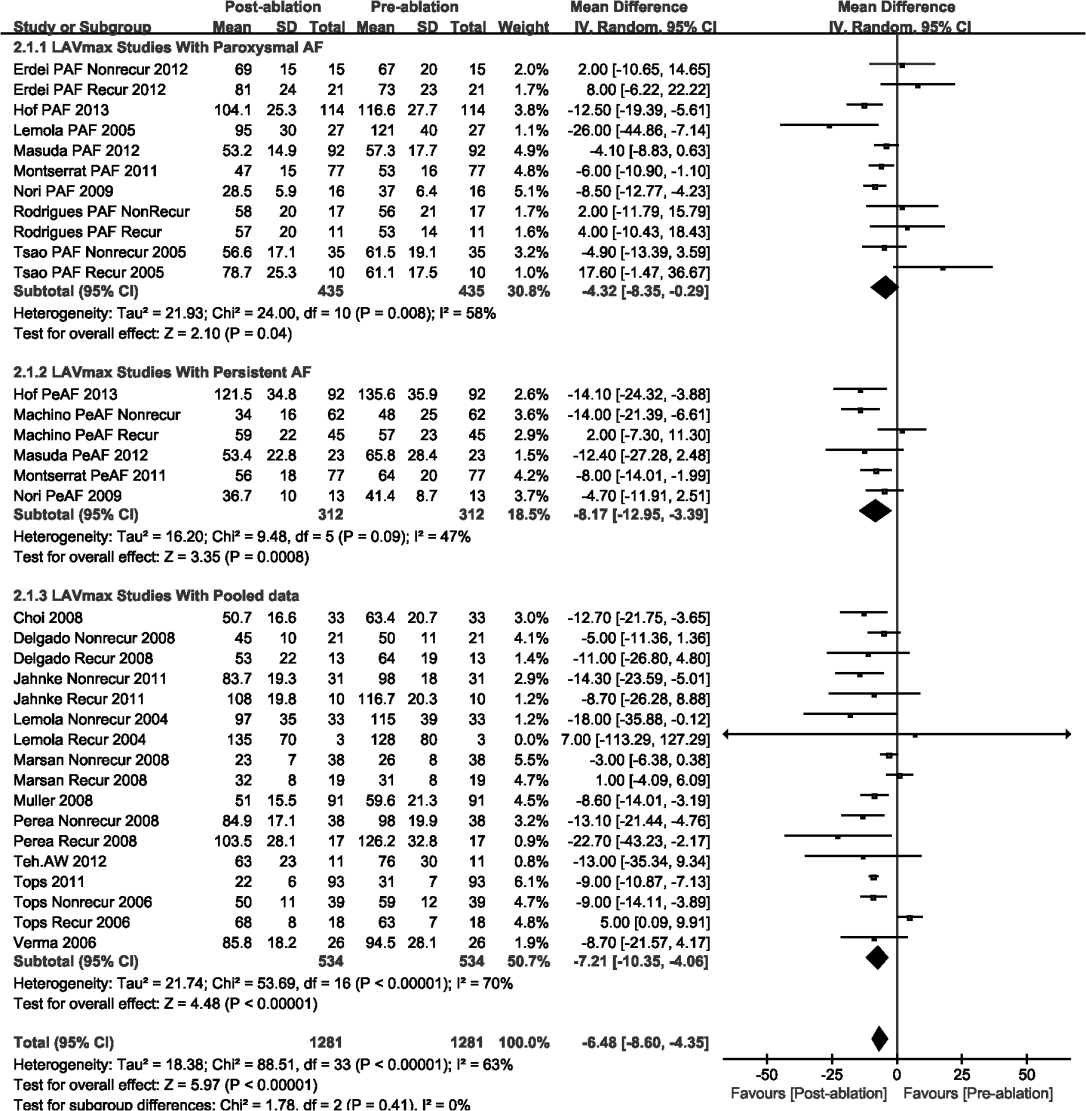
A forest plot of comparison: changes in maximum left atrial volume (LAVmax) pre-ablation and post-ablation.

**Fig 4:**
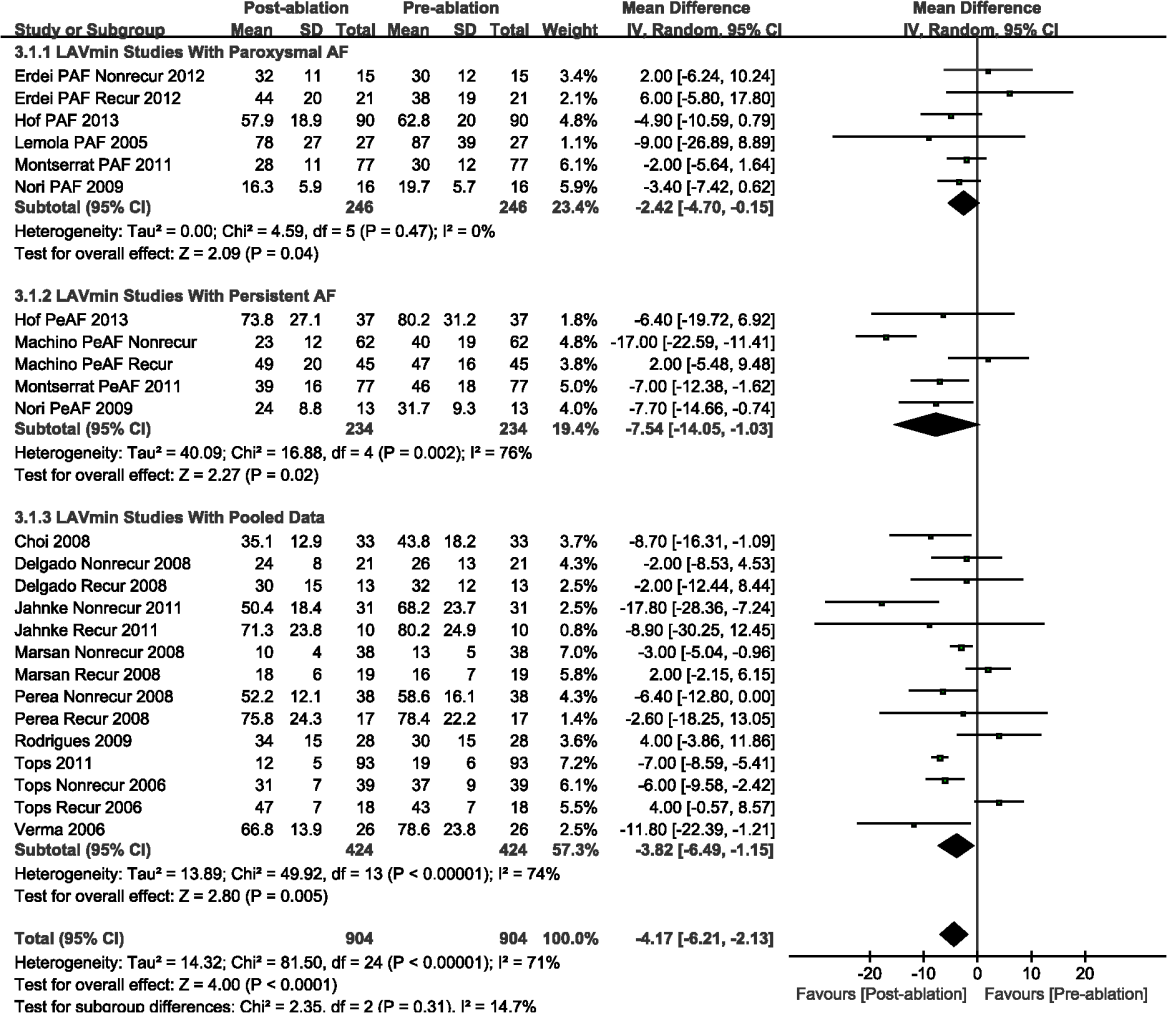
A forest plot of comparison: changes in minimum left atrial volume (LAVmin) pre-ablation and post-ablation.

The LAD (WMD, -0.91 mm; 95%CI, from -1.75 mm to -0.06 mm, *P* = 0.04; Fig 2), LAVmax (WMD, -6.48 mL; 95%CI, from -8.60 mL to -4.35 mL, *P* < 0.00001; Fig 3), and LAVmin (WMD, -4.17 mL; 95%CI, from -6.21 mL to -2.13 mL, *P* < 0.0001; Fig 4) were significantly decreased post-ablation, as compared with those pre-ablation. Nevertheless, a subgroup analysis was performed that was based on AF type; there were significant decreases in LA volumes (including LAVmax and LAVmin) for the AF patients. The LAD result indicated insignificant changes for patients with either paroxysmal or persistent AF (Figs 2–4).

**Fig 5:**
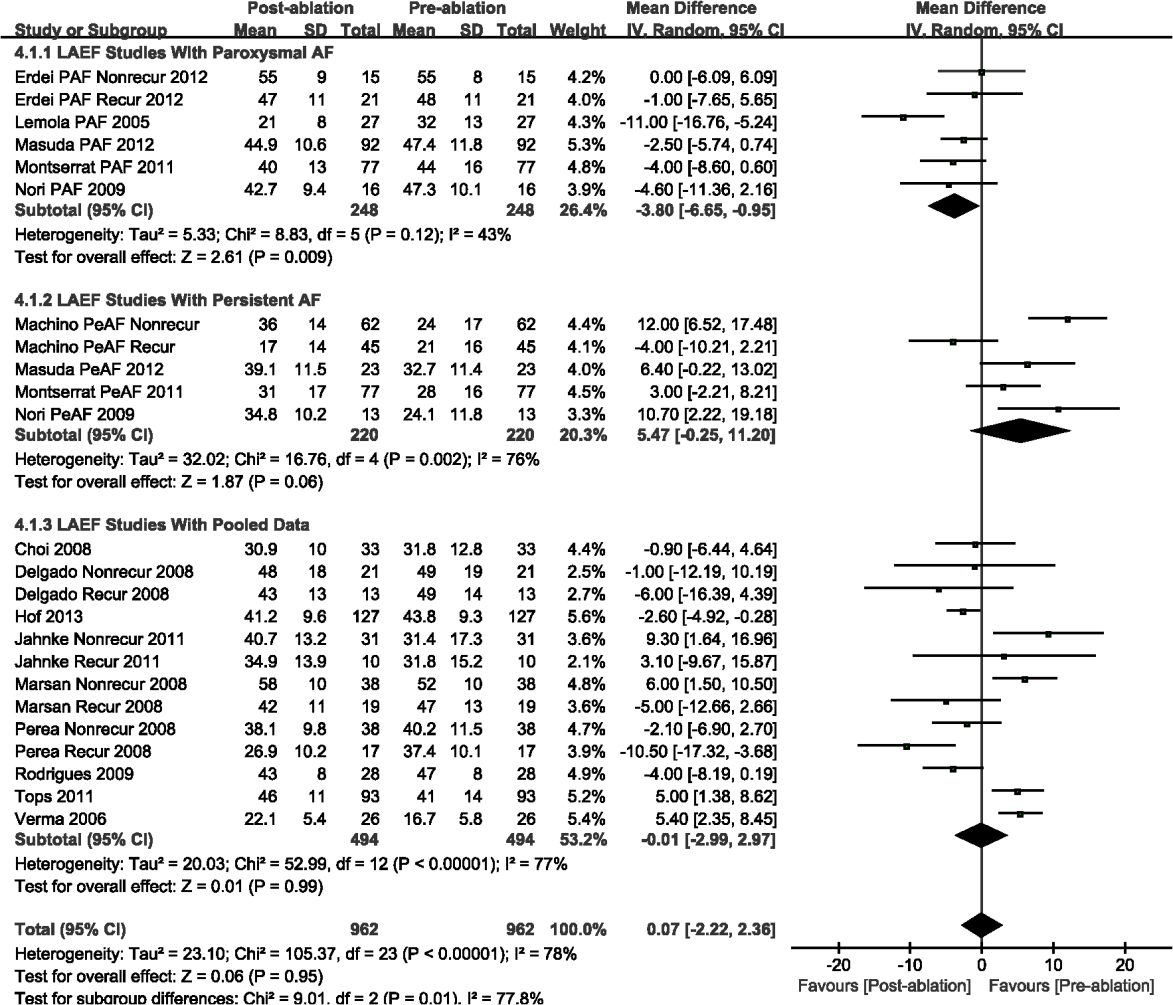
A forest plot of comparison: changes in left atrial ejective fraction (LAEF) pre-ablation and post-ablation.

**Fig 6:**
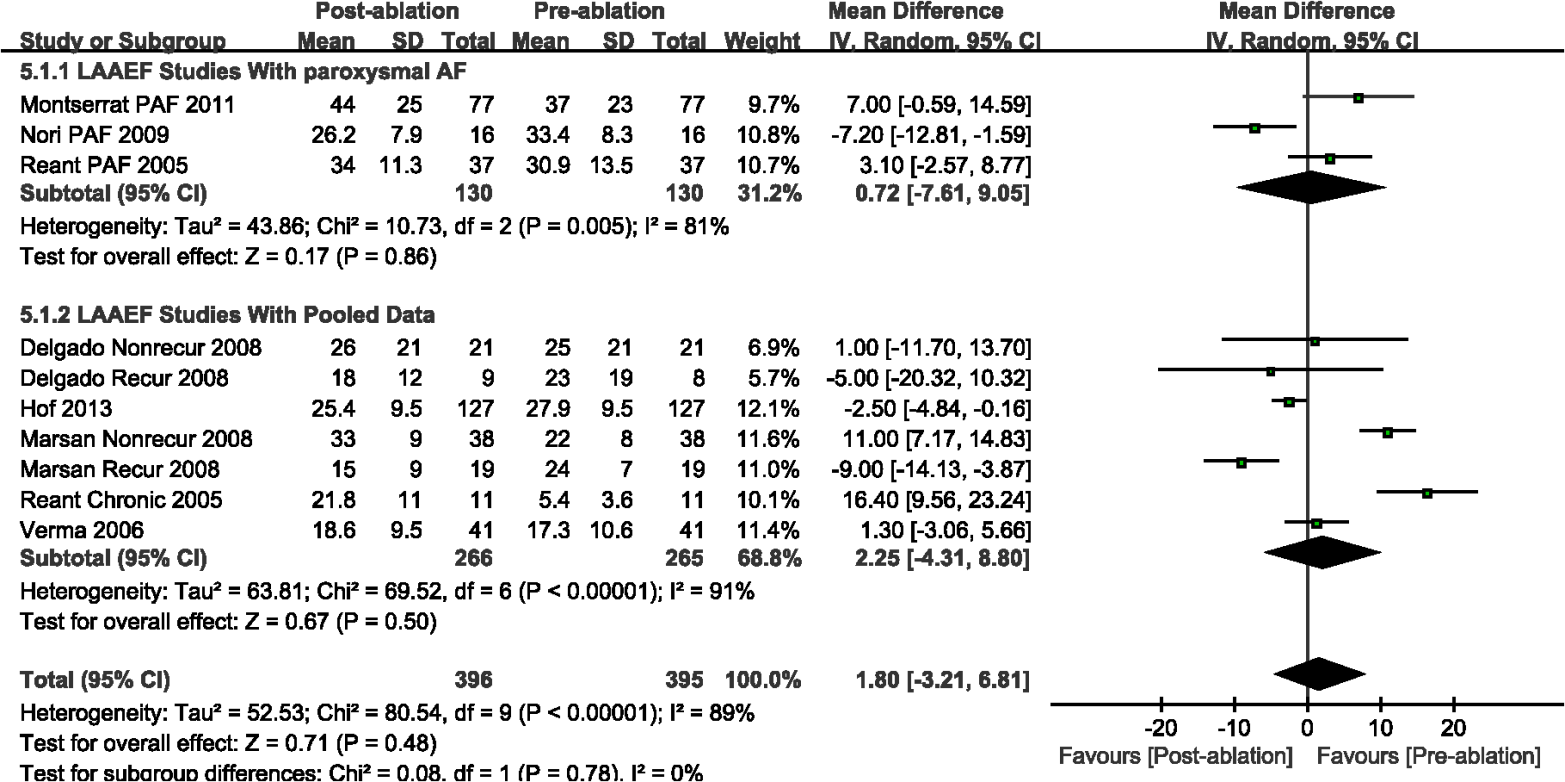
A forest plot of comparison: changes in left atrial active ejective fraction (LAAEF) pre-ablation and post-ablation.

**Fig 7:**
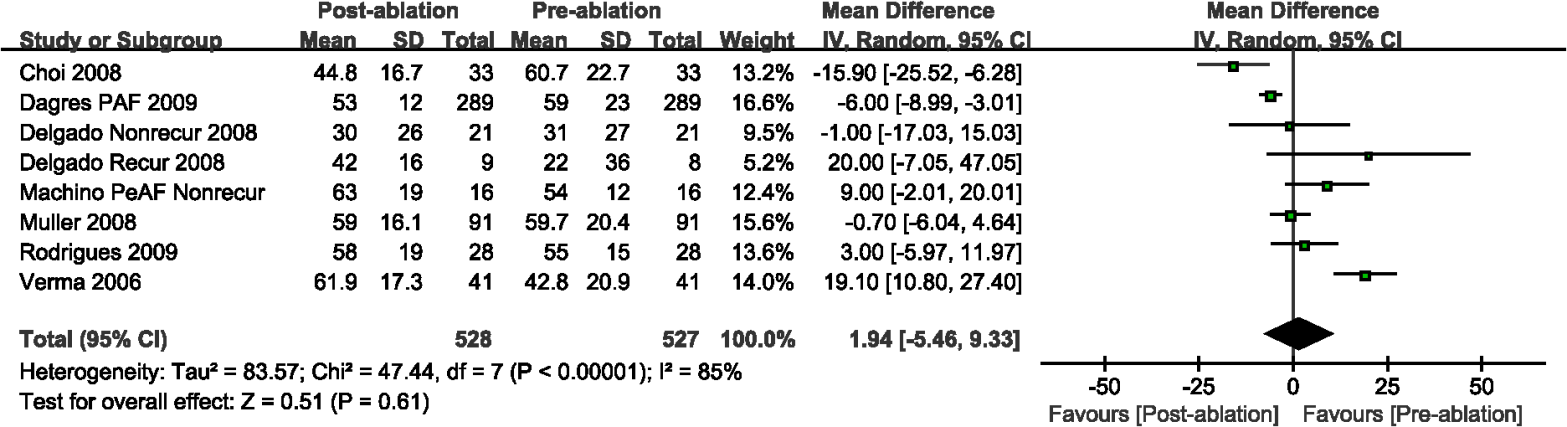
A forest plot of comparison: changes in A wave velocity pre-ablation and post-ablation.

**Fig 8:**
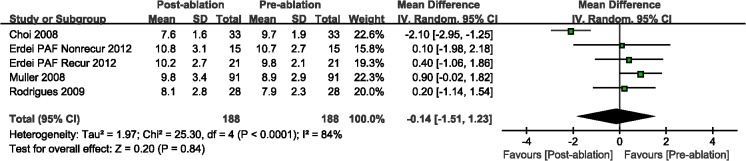
A forest plot of comparison: changes in A´ wave velocity pre-ablation and post-ablation.

Compared with the pre-ablation outcomes, we found no significant differences in LAEF (WMD, 0.07%; 95%CI, from -2.22% to 2.36%, *P* = 0.95; Fig 5) and LAAEF (WMD, -1.86%; 95%CI, from -3.92% to 7.63%, *P* = 0.48; Fig 6). Subsequently, we performed a subgroup analysis based on the AF type, and there were insignificant differences among those studies with either PAF or PeAF, except for LAEF with PAF (WMD, -3.80%; 95%CI, from -6.65% to -0.95%, *P* = 0.009; Fig 5). Finally, we analyzed the A wave velocity (A) and A' wave velocity (A'), and there were insignificant differences during follow-up imaging for CA treatment, as compared with pre-ablation (Figs 7 and 8).

Subsequently, we implemented a subgroup analysis on the basis of AF recurrence. There were significant decreased in LAD (WMD, -1.63 mm; 95%CI, from -3.01 mm to -0.24 mm, *P* = 0.02, S1 Fig), LAVmax (WMD, -7.53 mL; 95%CI, from -11.09 mL to -3.97 mL, *P* < 0.0001, S2 Fig), and LAVmin (WMD, -6.73 mL; 95%CI, from -11.07 mL to -2.39 mL, *P* = 0.002, S3 Fig) with no recurrence AF during post-ablation follow-up, but not those with AF recurrence, except for LAD with AF recurrence (WMD, 2.25 mm; 95%CI, from 0.29 mm to 4.21 mm, *P* = 0.02, S1 Fig). The LAEF (WMD, -4.60%; 95%CI, from -7.91% to -1.29%, *P* = 0.006, S4 Fig) and LAAEF (WMD, -8.60%; 95%CI, from -13.46% to -3.74%, *P* = 0.0005, S5 Fig) were decreased significantly in patients with recurrence AF after CA during follow-up, however, there were insignificant changes between those with no recurrence AF.

### Heterogeneity Analysis

After performing a heterogeneity test, the existence of heterogeneity among those studies should not be ignored. To demonstrate the origin of the heterogeneity, a meta-regression analysis and sensitivity analysis were performed. Heterogeneity was significant in spite which individual study was excluded. As previously illustrated, a subgroup analysis was performed; each outcome was analyzed based on the AF type (PAF or PeAF). A random effects model was used to combine the effect size because significant heterogeneity was shown as an all-total consequence.

Subsequently, a sensitivity analysis that was on the basis of image modalities, the LAVmax (WMD, -9.31 mL; 95%CI, from -12.45 mL to -6.16 mL, *P* < 0.00001, S7 Fig) and LAVmin (WMD, -6.07 mL; 95%CI, from -8.49 mL to -3.65 mL, *P* < 0.00001, S8 Fig) remained significant decreased at post-ablation which detected by cardiac magnetic resonance imaging (CMR) and/or Computed Tomography (CT). However, the LAD (WMD, -0.77 mm; 95%CI, from -2.87 mm to 1.33 mm, *P* = 0.47, S6 Fig), LAEF (WMD, -0.28%; 95%CI, from -3.91% to 3.35%, *P* = 0.88, S9 Fig) and LAAEF (WMD, -2.47%; 95%CI, from -6.30% to 1.36%, *P* = 0.21, S10 Fig) were not significant change during follow-up after catheter ablation treatment. And then, we found that the LAVmax (WMD, -7.08 mL; 95%CI, from -12.52 mL to -1.64 mL, *P* = 0.01, S7 Fig), LAVmin (WMD, -4.07 mL; 95%CI, from -7.29 mL to -0.84 mL, *P* = 0.01, S8 Fig) and LAEF (WMD, -5.72%; 95%CI, from -11.02% to -0.42%, *P* = 0.03, S9 Fig) were significant decrease in PAF; the LAV (LAVmax: WMD, -8.90 mL; 95%CI, from -15.28 mL to -2.53 mL, *P* = 0.006, S7 Fig; LAVmin: WMD, -7.42 mL; 95%CI, from -13.59 mL to -1.25 mL, *P* = 0.02, S8 Fig) were decreased significantly in PeAF, however, the LAEF (WMD, 8.03%; 95%CI, from 2.81% to 13.24%, *P* = 0.003, S9 Fig) was increased significantly in PeAF.

According to a sensitivity analysis that was based on a follow-up of >6 months, there were significant decreases in only LAV after catheter ablation therapy, including LAVmax (WMD, -6.07 mL; 95%CI, from -8.76 mL to -3.38 mL, *P* < 0.00001, S12 Fig) and LAVmin (WMD, -3.91 mL; 95%CI, from -6.62 mL to -1.20 mL, *P* = 0.005; S13 Fig). However, the LAD, LAEF, and LAAEF did not significantly change during follow-up after ablation treatment (WMD, -0.81 mm; 95%CI, from -1.68 mm to 0.06 mm, P = 0.07; WMD, 0.05%; 95%CI, from -2.67% to 2.77%, *P* = 0.97; WMD, 2.89%; 95%CI, from -2.42% to 8.20%, *P* = 0.29; respectively, S11, S14 and S15 Figs).

A sensitivity analysis was performed based on a follow-up of >12 months. After ablation therapy, the LAVmax (WMD, -7.83 mL; 95%CI, from -11.65 mL to -4.01 mL, *P* < 0.0001, S17 Fig) and LAVmin (WMD, -5.90 mL; 95%CI, from -9.77 mL to -2.03 mL, *P* = 0.003, S18 Fig) were significantly decreased; however, the LAD and LAEF did not significantly change (WMD, -0.36 mm; 95%CI, from -1.53 mm to 0.81 mm, *P* = 0.55; WMD, 0.80%; 95%CI, from -3.03% to 4.63%, *P* = 0.68; respectively, S16 and S19 Figs).

Subsequently, a meta-regression analysis was performed to determine the heterogeneity origin. However, we did not find any factors that contributed to the heterogeneity.

### Publication Bias Analysis

Egger’s test and Begg’s funnel plot were used to evaluate publication bias. There were no significant risks of publication bias according to an analysis using Stata 12.0 (the *P* value for each test was >0.05; Table 3). The funnel plot was generally symmetrical, and it indicated that the publication bias for the studies was controlled.

**Table 3:** Assessment of publication bias with Stata 12.0 for each primary outcome.

Primary Outcome	Begg’s Test (P value)	Egger’s Test (P value)
LAD	0.921	0.636
LAVmax	0.767	0.832
LAVmin	0.726	0.670
LAEF	0.785	0.948
LAAEF	1.000	0.605
A Wave	0.536	0.205
A' Wave	0.086	0.117

Abbreviations as previously detailed.

## Discussion

In the present review, we found that the LA volumes and LAD were significantly decreased after CA therapy during follow-up imaging. Nonetheless, we did not find any significant changes in LA function (included LAEF and LAAEF) after ablation treatment during follow-up imaging. Furthermore, there were significant decreases in the LA volumes and LAEF with paroxysmal AF after CA treatment. However, we did not find any significant changes in outcomes, as previously detailed, for persistent AF after ablation therapy, except for LA volumes.

CA is a therapeutic method for terminating the underlying electrophysiological mechanism of AF. The substrate and trigger foci are isolated by freezing (cryoablation) or radiofrequency energy and then terminate the electrical conduction from the pulmonary vein (PV) to LA. Currently, CA is approved by the Food and Drug Administration (FDA) for managing paroxysmal AF. Although this practical strategy is also used for managing non-paroxysmal AF, unfortunately, it is not yet approved by the FDA [2, 35]. The resumption of a sinus rhythm with CA is a perfect consequence, but the amount of LA scarring produced by CA could influence LA structural and functional remodeling, especially with repeated ablation. Structural remodeling includes increasing LA size and a change in LA strain. Several studies [10, 18, 19] reported that the enlargement could be reversed after successful ablation therapy that is defined as the maintenance of a sinus rhythm during follow-up [34]. Thus, LA reverse remodeling may become a robust sign of successful CA for patients with AF. Further studies should be conducted to evaluate the effects on LA function for patients with AF after a repeat ablation treatment.

There was a significant decrease in LAEF after CA treatment in studies with paroxysmal AF; however, we did not find similar outcomes in studies with persistent AF. Rodrigues et al. [17] reported a degradation in LAEF after CA for patients with paroxysmal AF at a follow-up duration of about 8 months after performing transthoracic echocardiography (TTE). Hof et al. [11] found a similar outcome using three-dimensional computed tomography (CT). However, Erdei et al. [10] and Machino-Ohtsuka et al. [13] described that the LAEF was preserved and even increased in patients without an AF recurrence at a follow-up of >12 months; however, it had decreased in AF recurrence patients after TTE and CMR. Why did this phenomenon occur in these studies? Several reasons for this variance should be considered, including the follow-up duration after CA therapy; the chronicity of AF; the different clinical outcomes; and the different degrees of tissue damage related to the different ablation strategies, tools, or both.

More consideration should be given to the follow-up duration regarding studying LA function and size. Studies with a long follow-up (not less than 12 months) [12, 13, 19] have illustrated significant increases in LAEF after ablation treatment in AF patients; however, insignificant changes in LAEF with paroxysmal AF in 3 months follow-up [14, 16]. Further, a sensitivity analysis based on the follow-up duration was not persuasive because of a lack of detailed individual patient data. McGann et al. [36] reported that the quantification and detection of left atrial wall scarring would be applicable 3 months after CA in patients with AF. As we known, there is a phenomenon of atrial “stunning” during in 1 month follow-up after catheter ablation therapy, and either the LA size or the LA function is unstable change. After the “blanking period” (about 3 months), which the rate of recurrence AF is highest [37], the quantification and detection of LA size and function is more credible and accurate. Therefore, it is important that a longer follow-up duration should be performed for evaluating LA function. Moreover, because the LA function was assessed using only the sinus rhythm (SR), it is difficult to evaluate LA function in permanent AF patients.

In addition, the imaging technique is another important factor. Many different methods were performed in the studies, including TTE [9, 10, 13–15, 17–20, 22, 24, 26–32], CMR [11, 12, 16, 25, 33], and CT [14, 21, 23, 27]. As an established method in cardiac imaging modalities, TTE can identify the size of each chamber, as well as the ejection fraction of the LA and left ventricle (LV). However, a limitation occurs if patients are obese and have serious obstructive pulmonary disease with poor acoustic windows [38]. Multidetector Computed Tomography (MDCT) has a prominent temporal and spatial resolution for measuring LA volumes. CMR can concurrently discover pre-ablation fibrosis and post-ablation scar tissue and measure PV anatomy in patients who undergo CA therapy [39]. As we known, different analytical methods or image tools would obtain different results. Compare to TTE, using CMR and CT have a prominent temporal and spatial resolution for measuring LA volumes and EF, the results of CMR or CT should be more accurate than that of TEE. Due to this important issue, we performed subgroup analysis based on the variant methods of image. Subsequently, after excluding studies using TTE, only 9 studies (enrolled 635 patients) [11, 12, 14, 16, 21, 23, 25, 27, 33] were included in the subgroup analysis on the basis of detecting by CMR and/or CT. The LA volumes significantly decreased, LAEF/LAAEF insignificant changed. The explanations for this phenomenon as follow. First, CMR and CT/MDCT are more accuracy and improve reproducibility in measurement of LA volumes and functions compare to TTE. Second, the numbers of included studies were decreased, and then it may influence the pooled data. Therefore, compared with MDCT and CMR, TTE may underestimate the true LA size and function.

Nonetheless, there is no gold standard for measuring LA function. In the present review, the LAEF was used to define LA function in 15 studies [10–17, 19, 23, 27–29, 31, 33], and only 6 studies used LAAEF to define LA function [11, 15, 16, 24, 29, 31]. Furthermore, A wave velocity [9, 13, 17, 27–29, 32] and A' wave velocity [10, 17, 28, 32] were used to define LA contractile function. The A wave velocity involves the peak velocities of the late transmitral flow, as measured by pulsed-wave DE, reflecting LA systolic function from hemodynamics, but it is not sensitive because it can be affected by the LV diastolic function and preload. However, compared with the A wave velocity, the A' wave velocity, as detailed previously, is an easy and effective means to assess LA systolic function from tissue motion because it is correlated with changes in the LA systolic area and volume [8]. Therefore, further studies should be conducted to assess this method for evaluating LA function.

Beyond these, the treatment strategy and energy of catheter ablation are another factor. In this meta-analysis, the majority of included studies were used RFCA, only one study [10] performed cryoablation. As we known, different treatment strategies, such as SPVI and PVAI, lead to different outcomes, and diverse ablation temperature and power resulted in different damages for atrium. The included studies used RFCA were set at a similar value of the ablation temperature and power, and therefore, the results have consistency and comparability. After excluding the study performed cryoablation, there were similar pooled data compare to previous detailed. Due to there was no more available data, further studies should be focused on evaluating the effects on LA function and size for patients with AF after cryoablation treatment.

Heterogeneity is an important issue for explaining the outcomes of this review, and significant heterogeneity was found in this meta-analysis. Subsequently, sensitivity analyses were performed, and heterogeneity was significant in spite which individual study was excluded. We did not find any contributing factor for the heterogeneity with a meta-regression. The quality of the included articles may be the origin of heterogeneity.

Moreover, our review had some limitations. First, we did not consider any randomized control trial in this meta-analysis; the sample sizes of the included studies were small, and most were single center and either a prospective or retrospective study that may have added potential biases to such studies. Second, it is difficult to draw decisive conclusions regarding LA functional change after ablation therapy, because of inconsistencies regarding individual patient data, the imaging method, and the follow-up duration. Third, although publication bias was not significant after performing an Egger’s test and a Begg’s funnel plot, the influence of bias in this article could not be thoroughly excluded, as only studies published in English were included. Forth, we have tried addressing an issue but indirectly regarding the effectiveness of CA for AF by looking at LA size and function, however, it is a pooled data and it has its own set of issues which precludes us from providing any more clarity. Moreover, another limitation is the lack of a gold standard to measure LA function among these involved studies. Currently, MDCT and CMR are considered relatively accurate methods for measuring LA function and size. Finally, although several studies reported that the LA volumes and sizes are predictors of AF recurrence after CA therapy [40–42], our review did not perform an analysis based on AF recurrence in different types of AF. Therefore, we do not know the relationship between AF recurrence and LA function/size among different types of AF.

In conclusion, With CA, LA volumes and LAD were decreased significantly in patients with AF; LAEF was not significant changes in patients with PeAF but decreased in those with PAF.

## Supporting Information

S1 Checklist The PRISMA Checklist. (DOC)

S1 Fig A forest plot of comparison: changes in left atrial diameter (LAD) pre-ablation and post-ablation on the basis of atrial fibrillation recurrence. (TIF)

S2 Fig A forest plot of comparison: changes in maximum left atrial volume (LAVmax) pre-ablation and post-ablation on the basis of atrial fibrillation recurrence. (TIF)

S3 Fig A forest plot of comparison: changes in minimum left atrial volume (LAVmin) pre-ablation and post-ablation on the basis of atrial fibrillation recurrence. (TIF)

S4 Fig A forest plot of comparison: changes in left atrial ejective fraction (LAEF) pre-ablation and post-ablation on the basis of atrial fibrillation recurrence. (TIF)

S5 Fig A forest plot of comparison: changes in left atrial active ejective fraction (LAAEF) pre-ablation and post-ablation on the basis of atrial fibrillation recurrence. (TIF)

S6 Fig A forest plot of comparison: changes in left atrial diameter (LAD) pre-ablation and post-ablation detected by cardiac magnetic resonance imaging and/or computed tomography. (TIF)

S7 Fig A forest plot of comparison: changes in maximum left atrial volume (LAVmax) pre-ablation and post-ablation detected by cardiac magnetic resonance imaging and/or computed tomography. (TIF)

S8 Fig A forest plot of comparison: changes in minimum left atrial volume (LAVmin) pre-ablation and post-ablation detected by cardiac magnetic resonance imaging and/or computed tomography. (TIF)

S9 Fig A forest plot of comparison: changes in left atrial ejective fraction (LAEF) pre-ablation and post-ablation detected by cardiac magnetic resonance imaging and/or computed tomography. (TIF)

S10 Fig A forest plot of comparison: changes in left atrial active ejective fraction (LAAEF) pre-ablation and post-ablation detected by cardiac magnetic resonance imaging and/or computed tomography. (TIF)

S11 Fig A forest plot of comparison: changes in left atrial diameter (LAD) pre-ablation and post-ablation during follow-up more than 6 months. (TIF)

S12 Fig A forest plot of comparison: changes in maximum left atrial volume (LAVmax) pre-ablation and post-ablation during follow-up more than 6 months. (TIF)

S13 Fig A forest plot of comparison: changes in minimum left atrial volume (LAVmin) pre-ablation and post-ablation during follow-up more than 6 months. (TIF)

S14 Fig A forest plot of comparison: changes in left atrial ejective fraction (LAEF) pre-ablation and post-ablation during follow-up more than 6 months. (TIF)

S15 Fig A forest plot of comparison: changes in left atrial active ejective fraction (LAAEF) pre-ablation and post-ablation during follow-up more than 6 months. (TIF)

S16 Fig A forest plot of comparison: changes in left atrial diameter (LAD) pre-ablation and post-ablation during follow-up more than 12 months. (TIF)

S17 Fig A forest plot of comparison: changes in maximum left atrial volume (LAVmax) pre-ablation and post-ablation during follow-up more than 12 months. (TIF)

S18 Fig A forest plot of comparison: changes in minimum left atrial volume (LAVmin) pre-ablation and post-ablation during follow-up more than 12 months. (TIF)

S19 Fig A forest plot of comparison: changes in left atrial ejective fraction (LAEF) pre-ablation and post-ablation during follow-up more than 12 months. (TIF)
