# Supplementary figures and images for: The Effect of Catheter Ablation on Left Atrial Size and Function for Patients with Atrial Fibrillation: An Updated Meta-Analysis
Source: PLoS One. 2015 Jul 6;10(7):e0129274. doi: 10.1371/journal.pone.0129274 (PMC4493108; doi:10.1371/journal.pone.0129274)

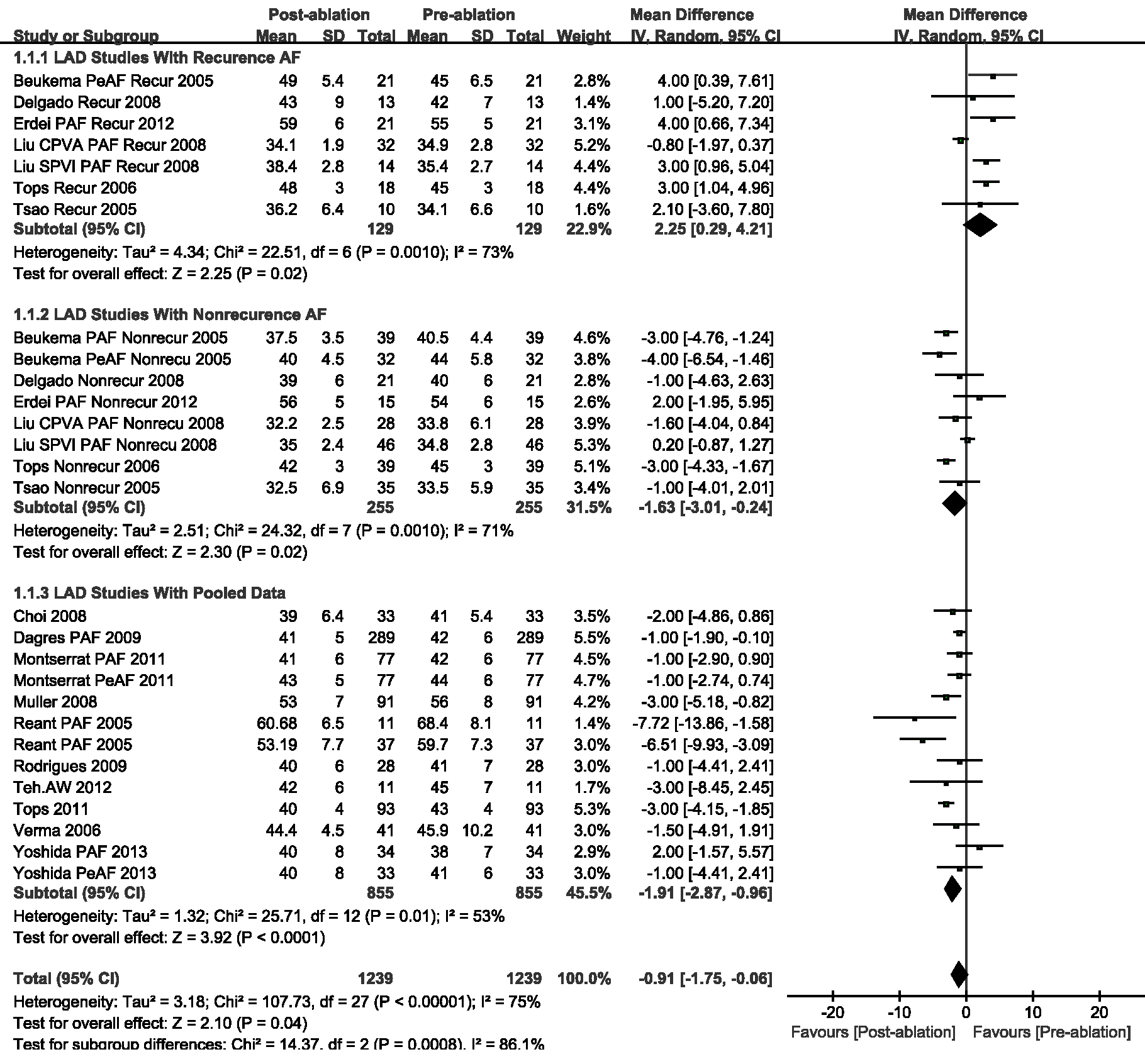

Supplement: S1 Fig — (TIF) [file pone.0129274.s002.tif]

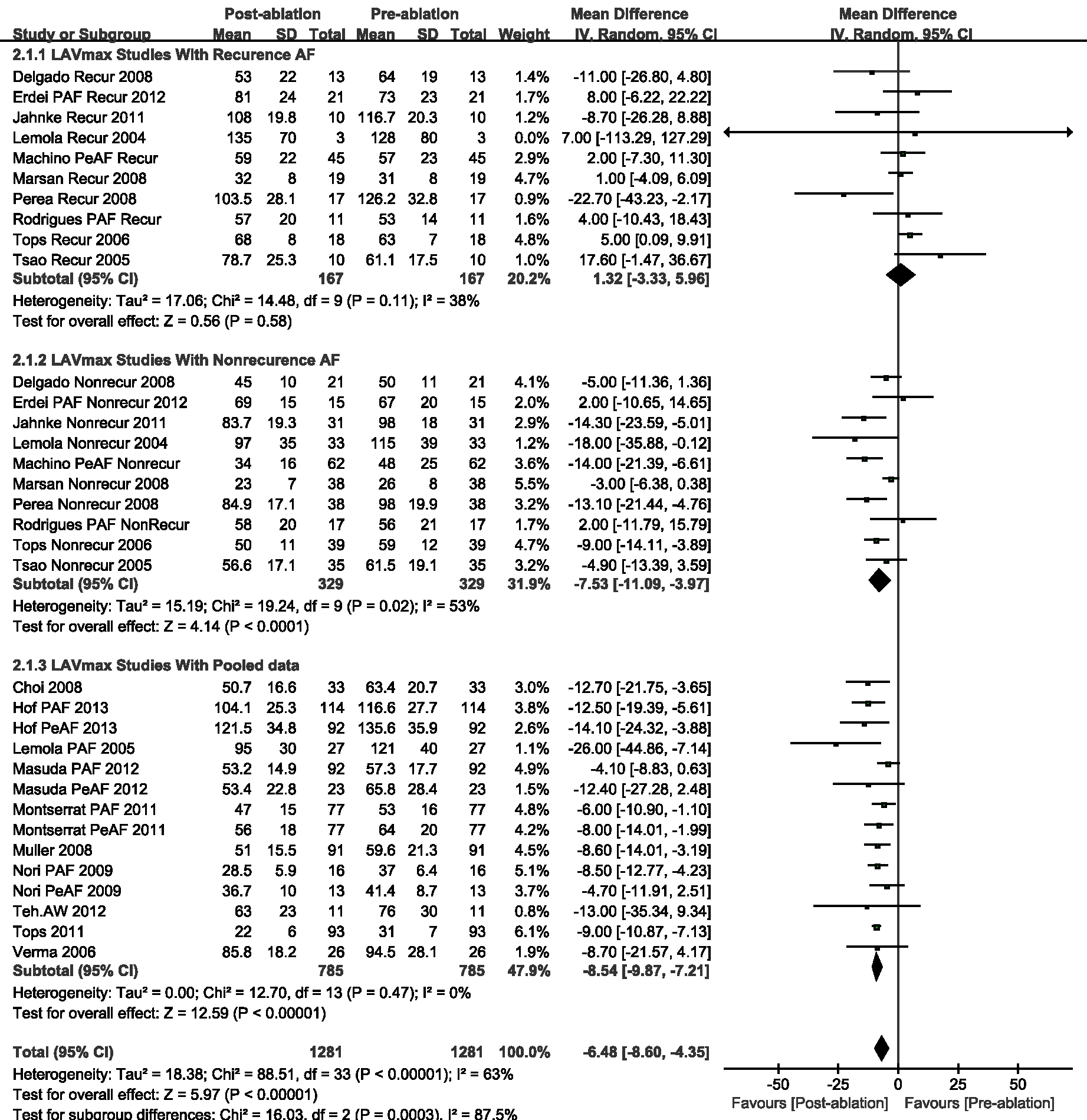

Supplement: S2 Fig — (TIF) [file pone.0129274.s003.tif]

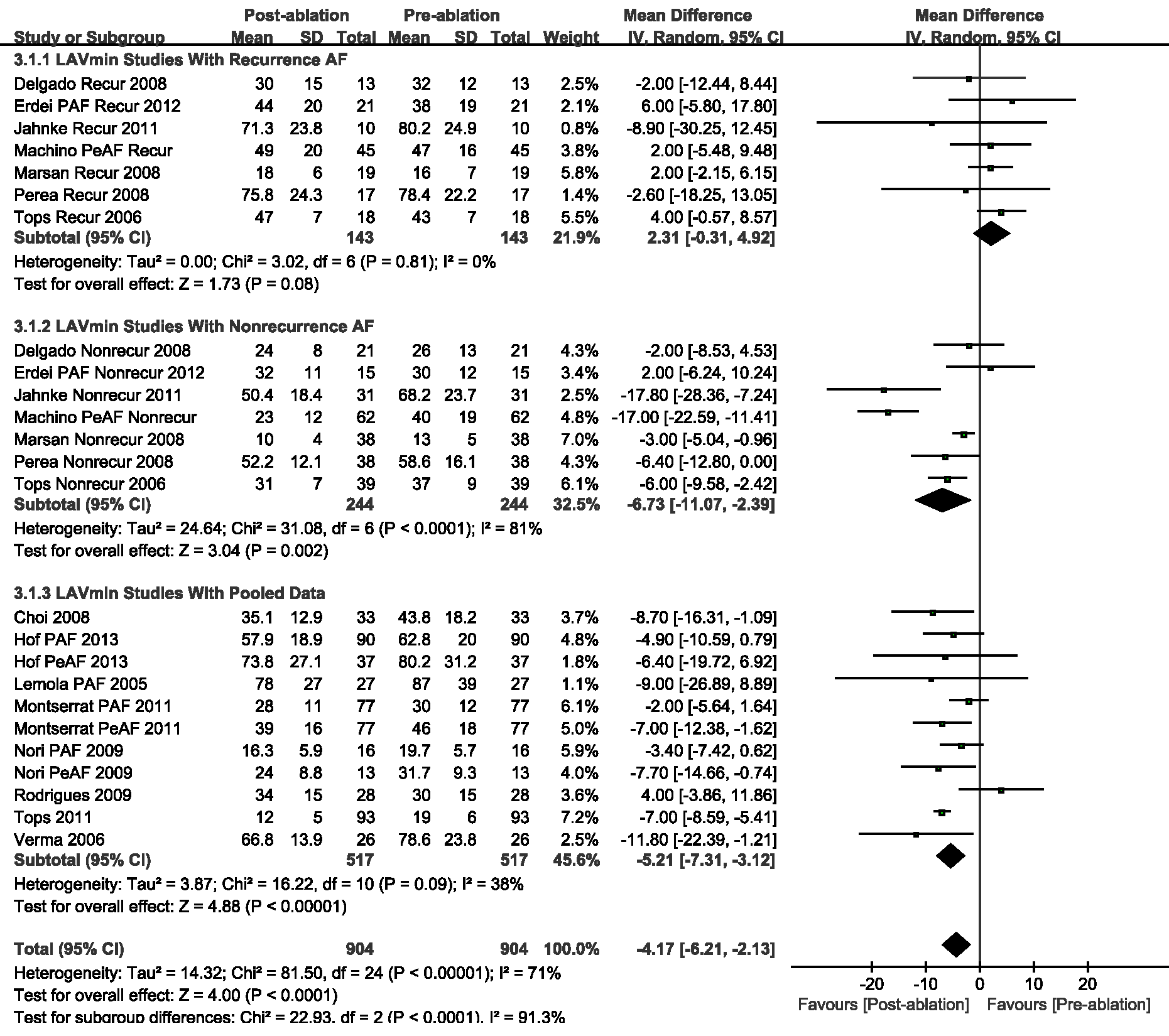

Supplement: S3 Fig — (TIF) [file pone.0129274.s004.tif]

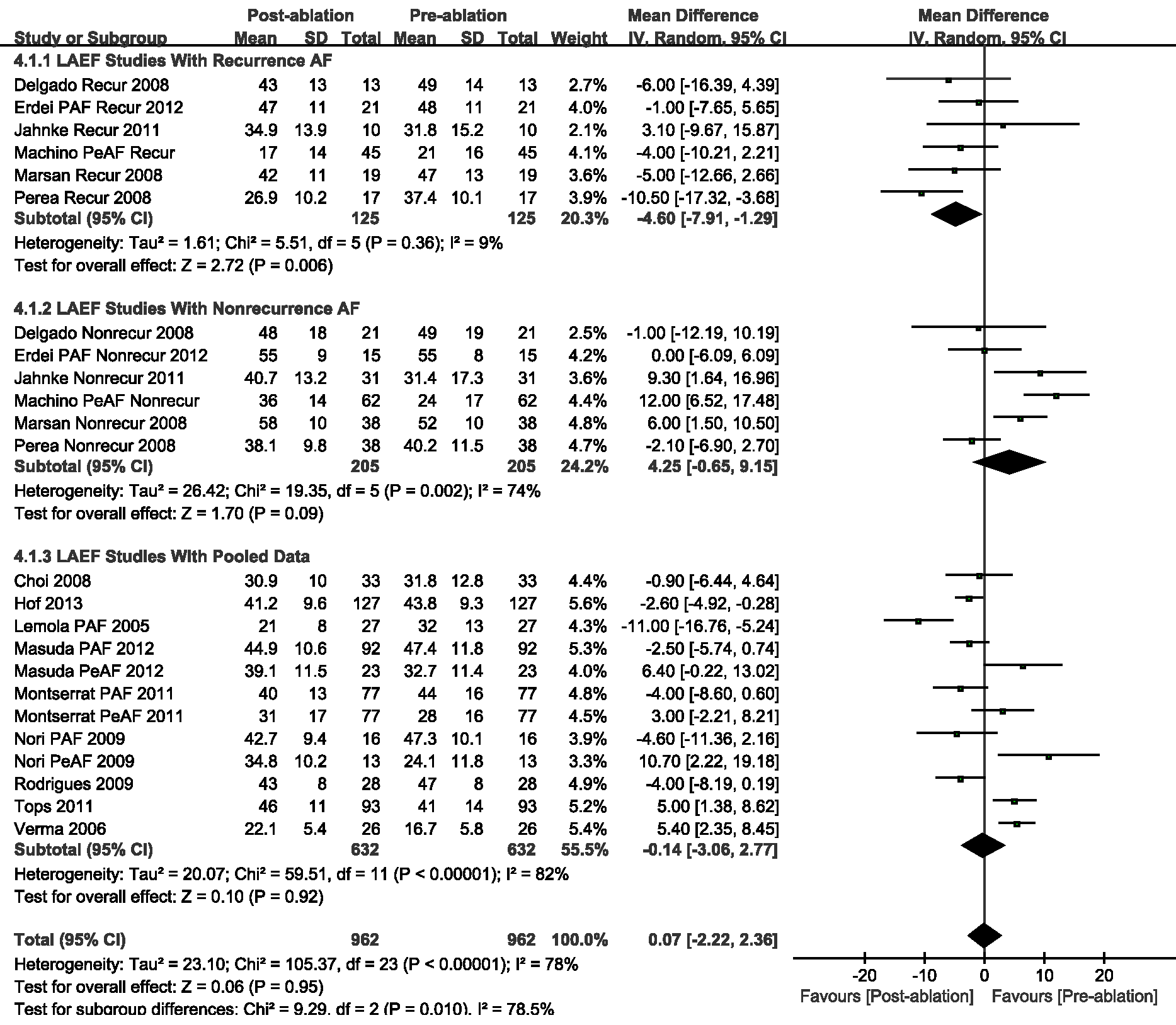

Supplement: S4 Fig — (TIF) [file pone.0129274.s005.tif]

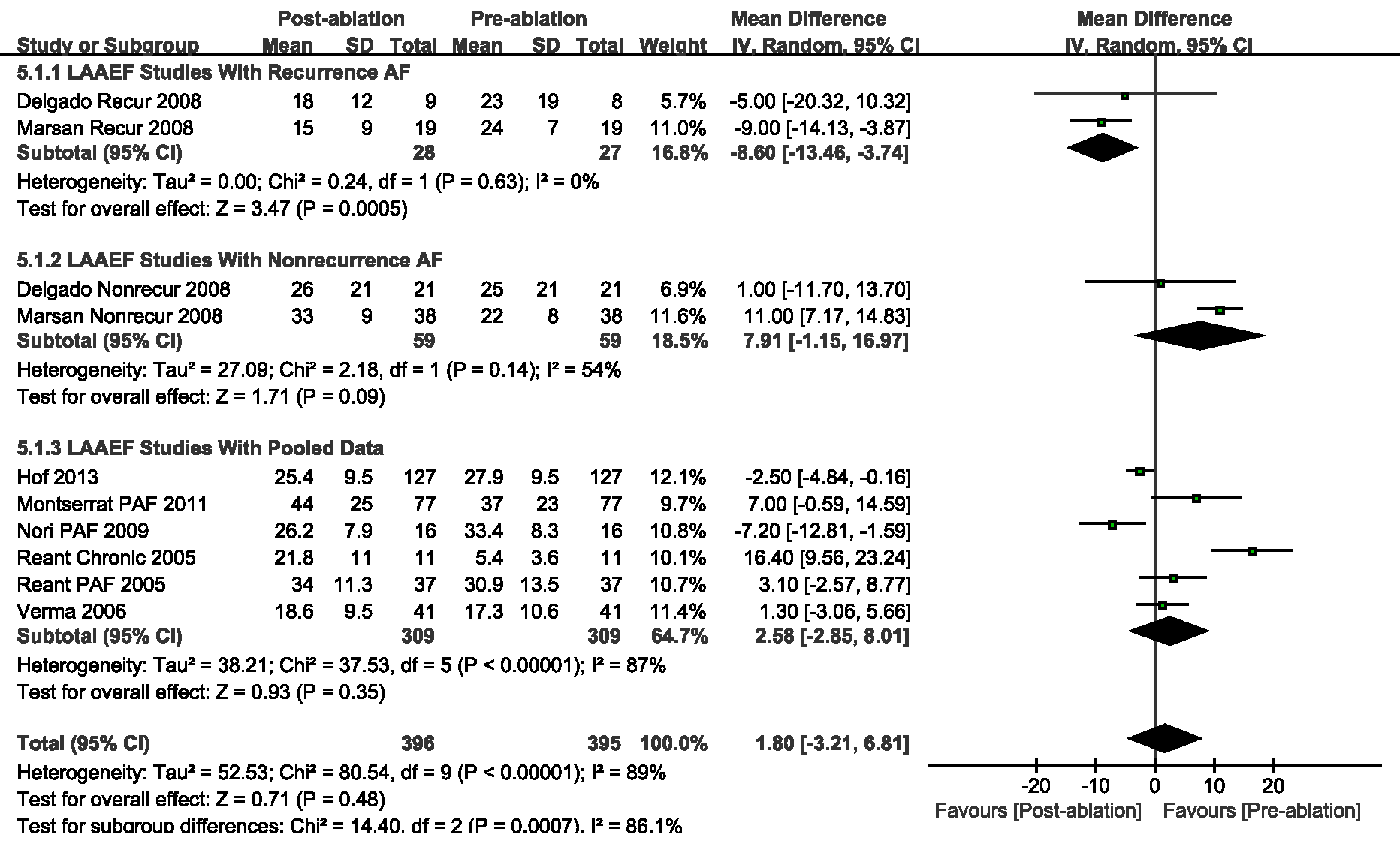

Supplement: S5 Fig — (TIF) [file pone.0129274.s006.tif]

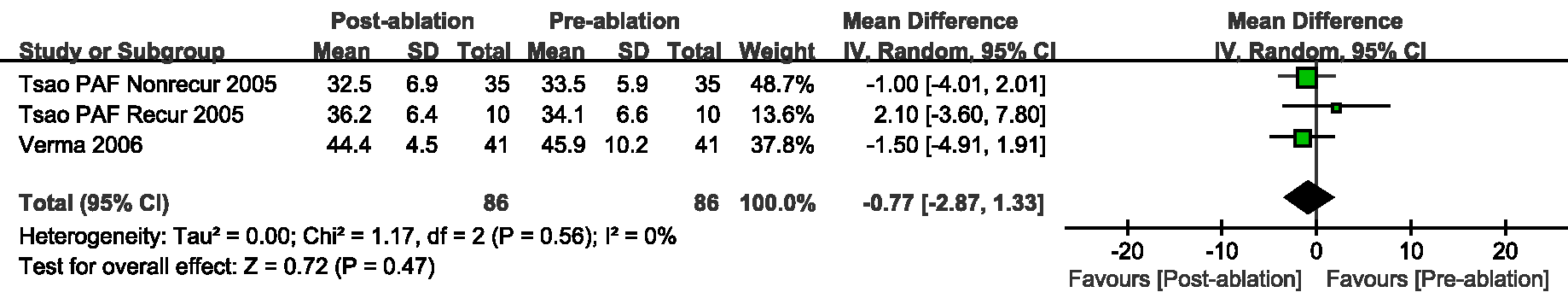

Supplement: S6 Fig — (TIF) [file pone.0129274.s007.tif]

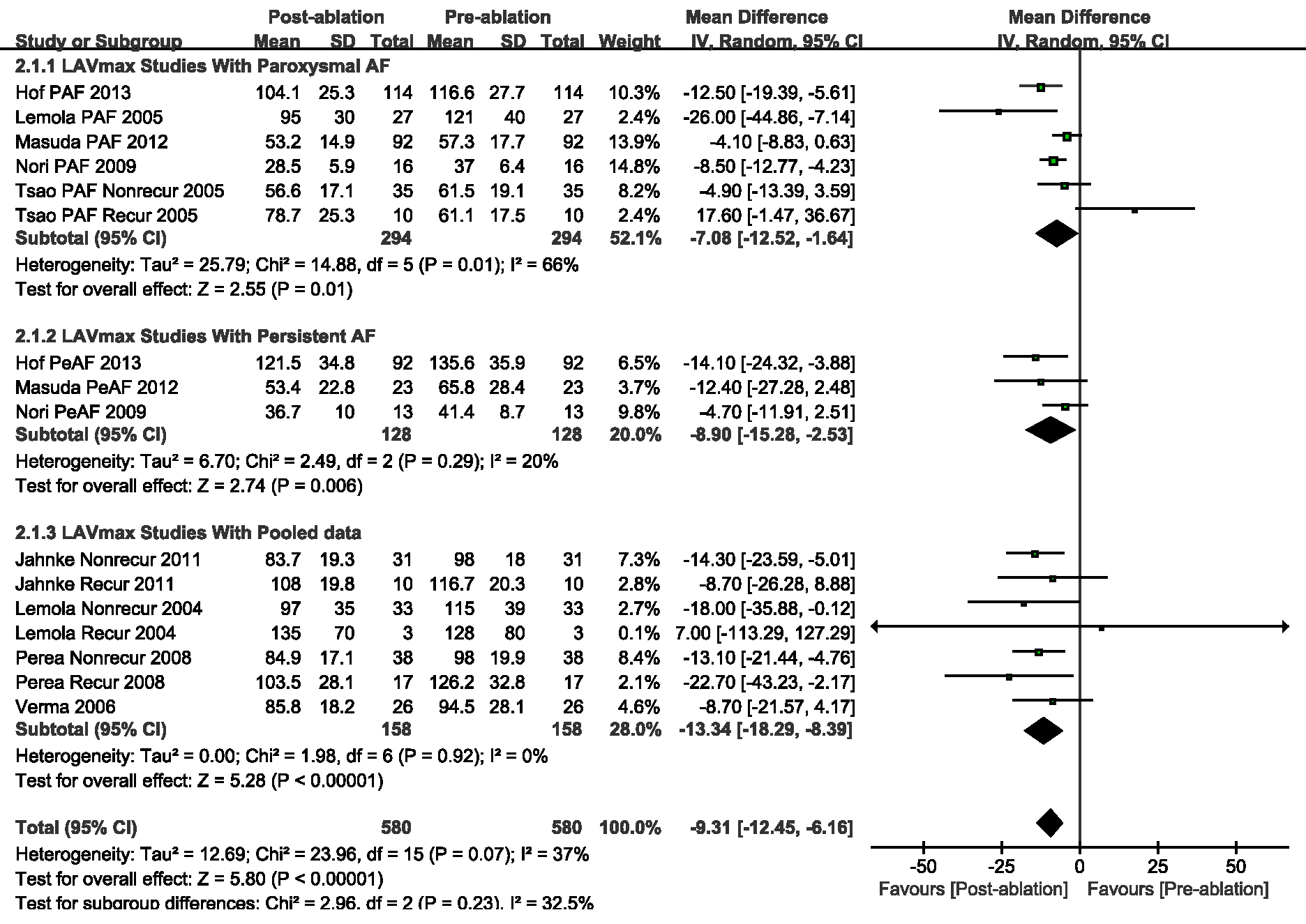

Supplement: S7 Fig — (TIF) [file pone.0129274.s008.tif]

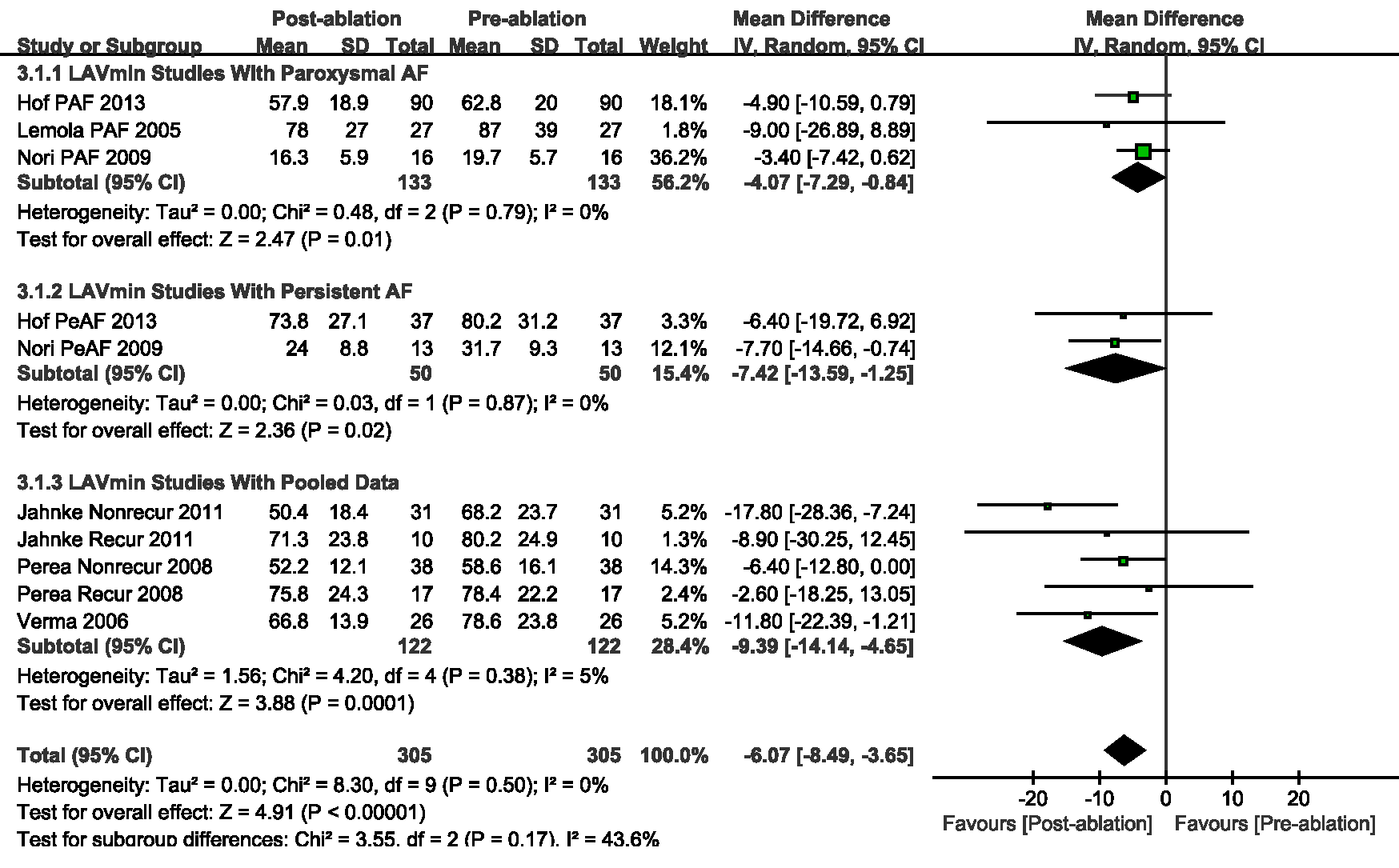

Supplement: S8 Fig — (TIF) [file pone.0129274.s009.tif]

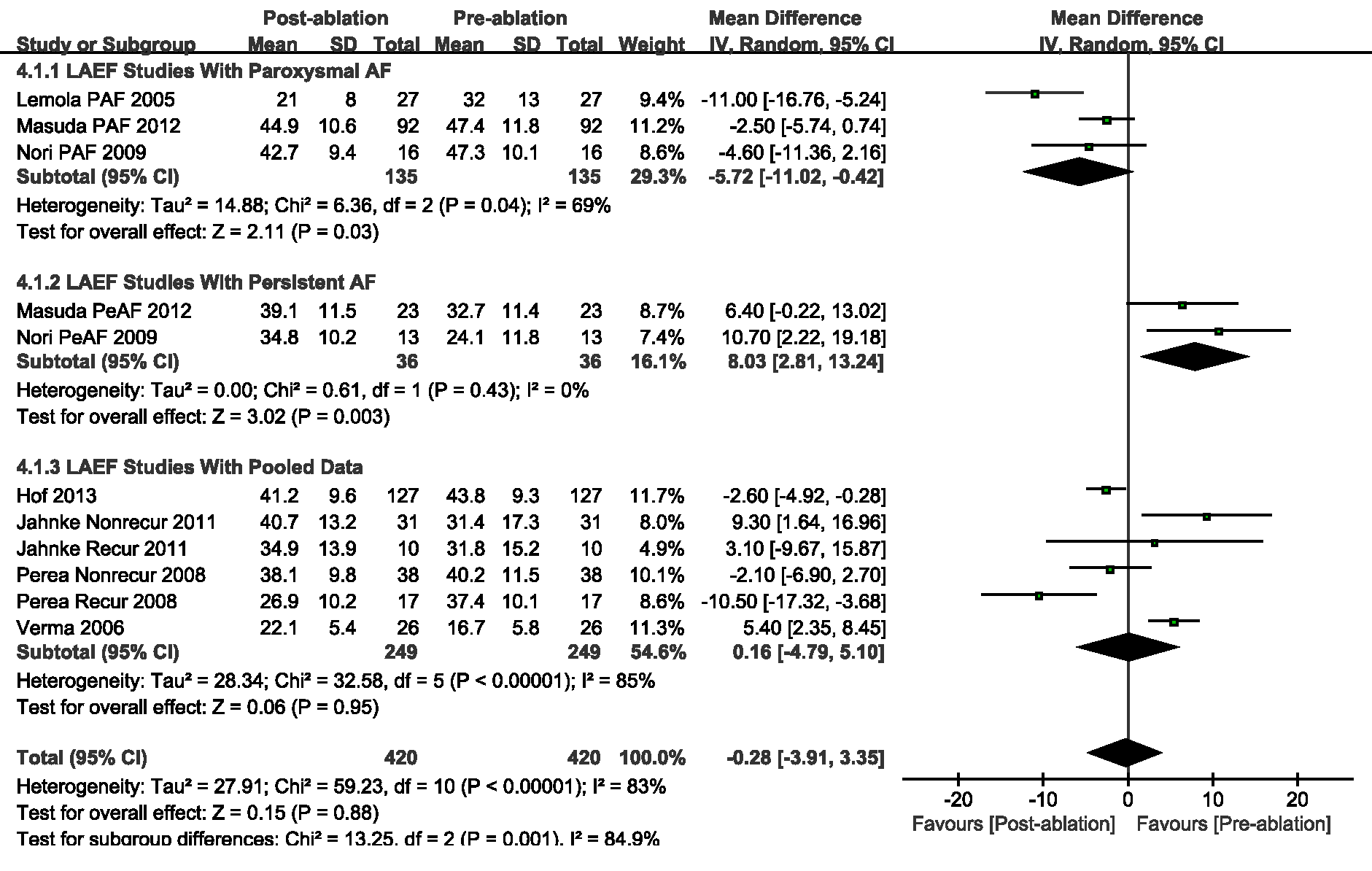

Supplement: S9 Fig — (TIF) [file pone.0129274.s010.tif]

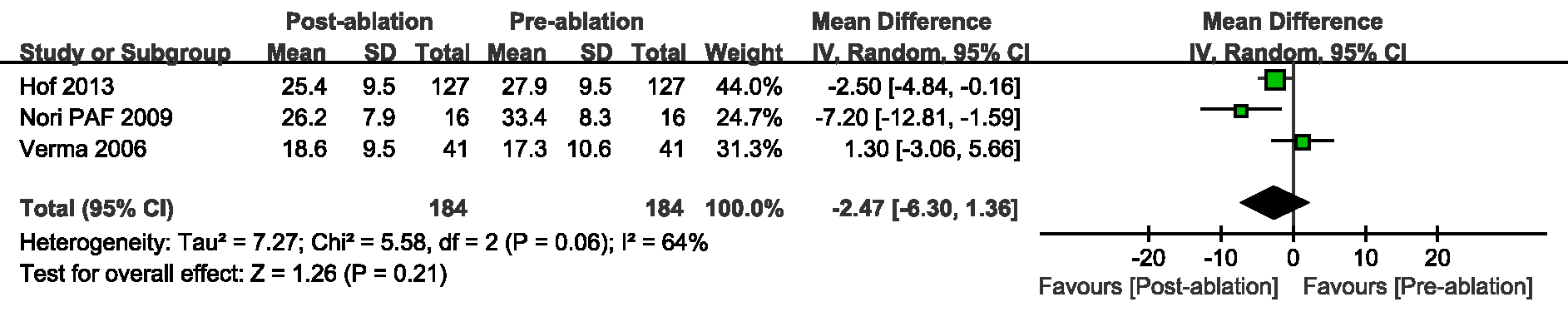

Supplement: S10 Fig — (TIF) [file pone.0129274.s011.tif]

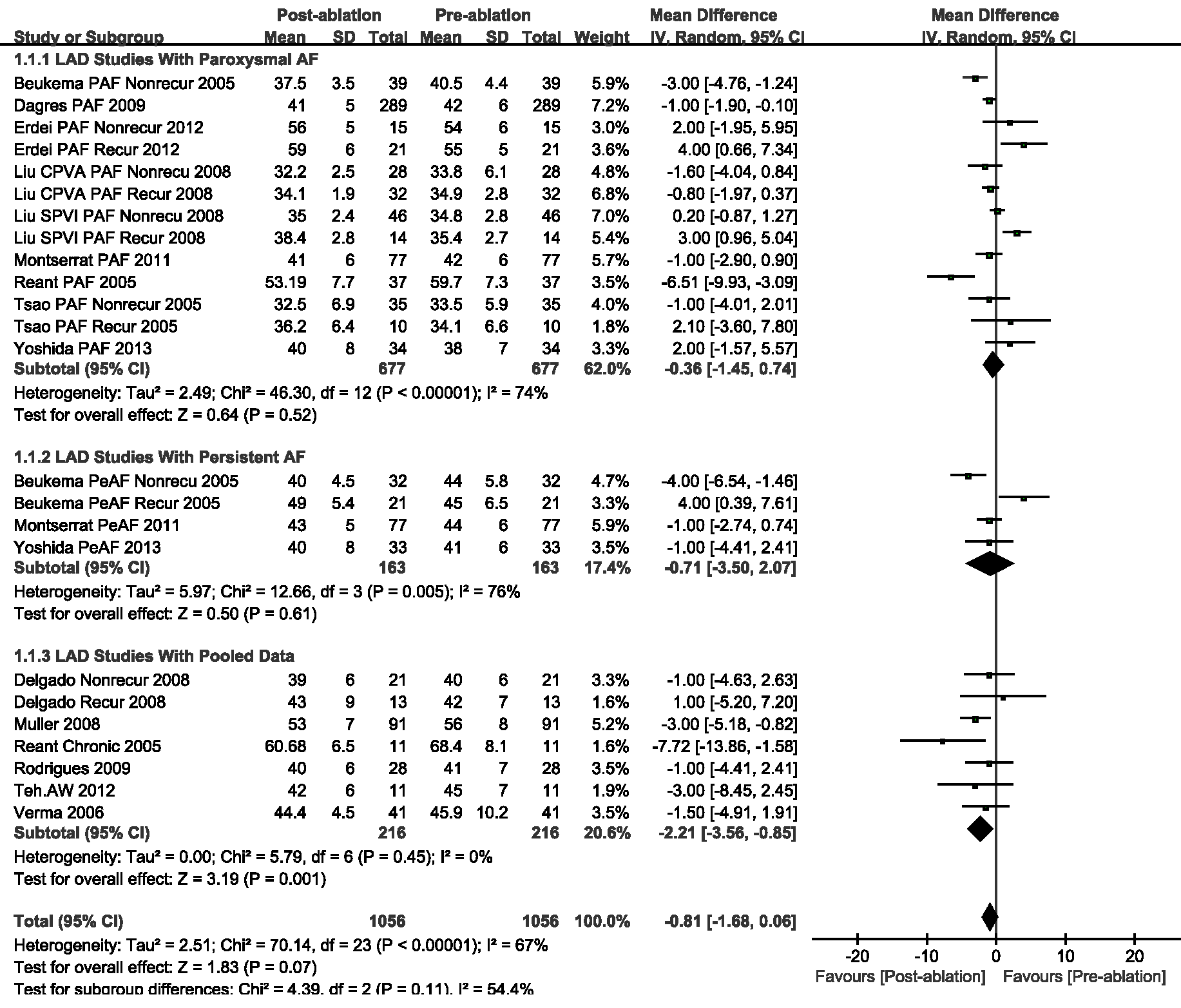

Supplement: S11 Fig — (TIF) [file pone.0129274.s012.tif]

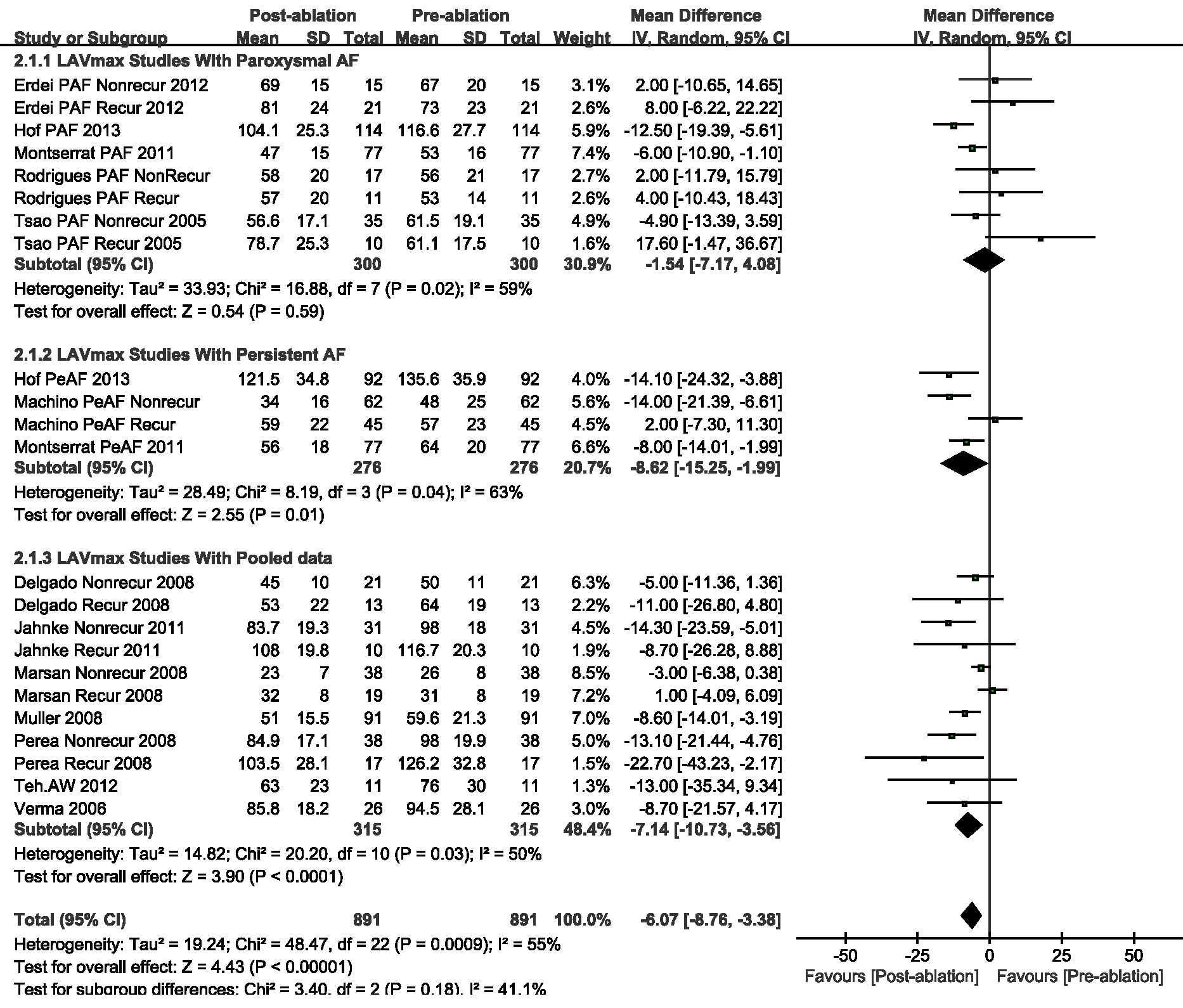

Supplement: S12 Fig — (TIF) [file pone.0129274.s013.tif]

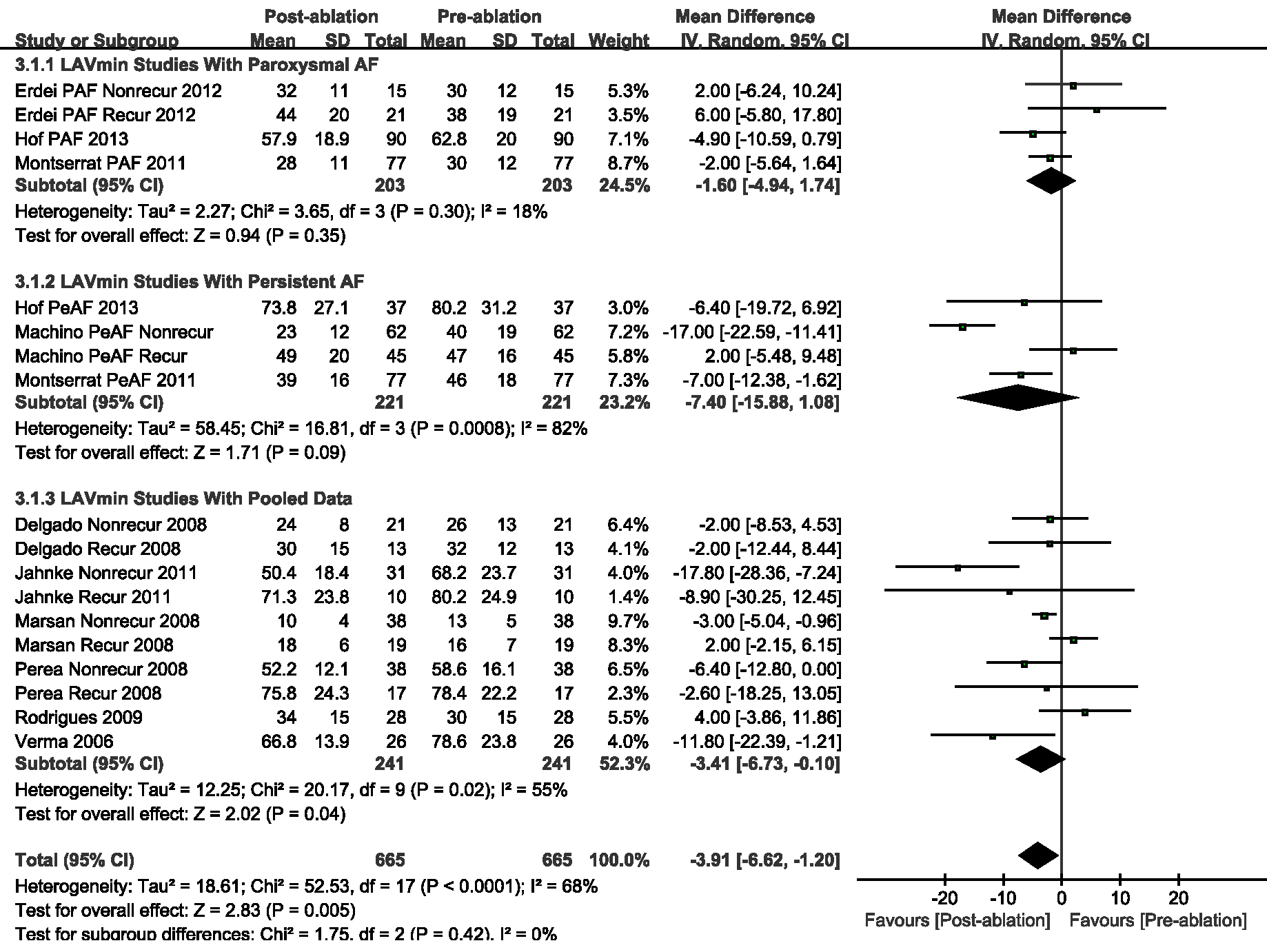

Supplement: S13 Fig — (TIF) [file pone.0129274.s014.tif]

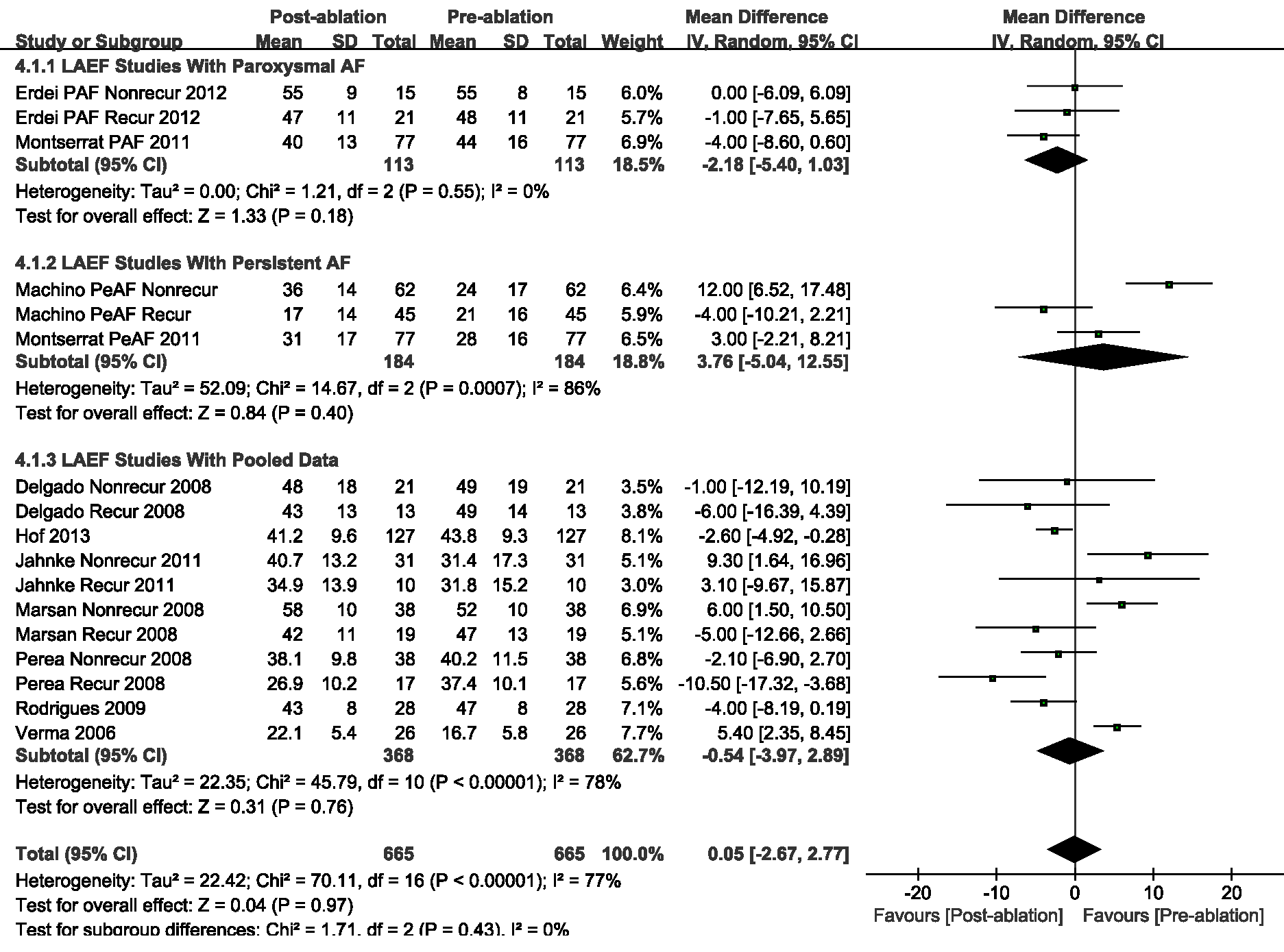

Supplement: S14 Fig — (TIF) [file pone.0129274.s015.tif]

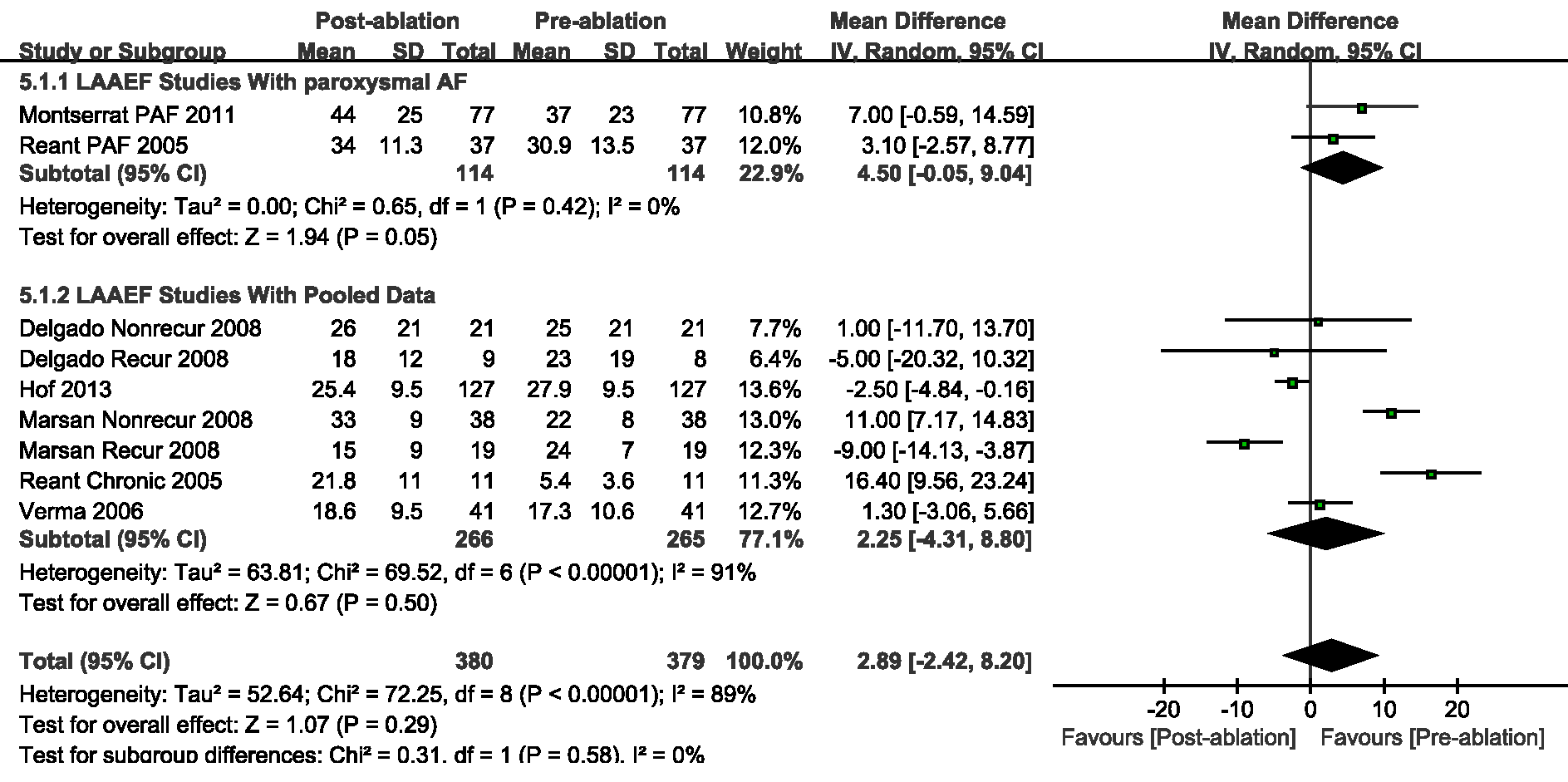

Supplement: S15 Fig — (TIF) [file pone.0129274.s016.tif]

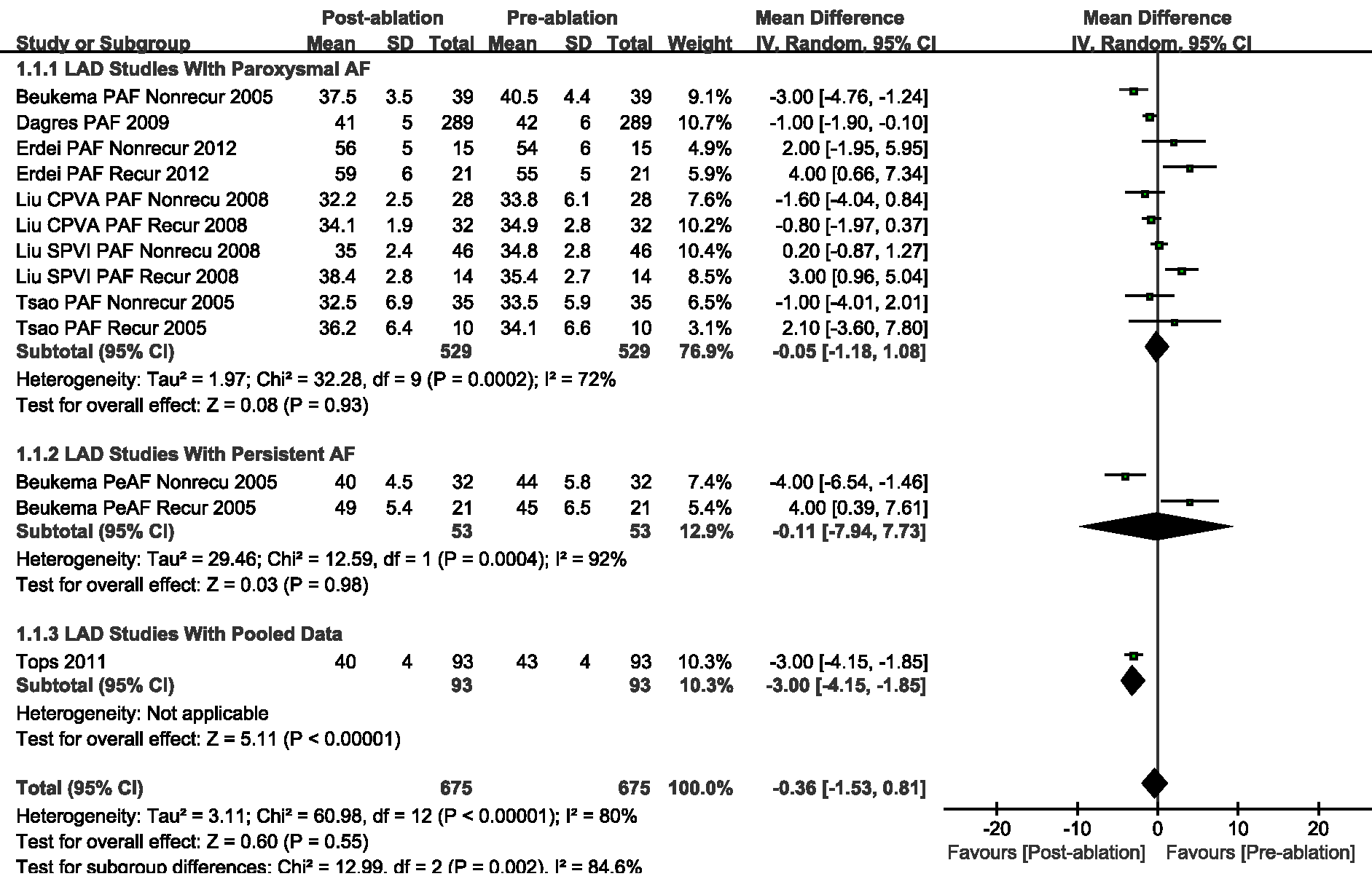

Supplement: S16 Fig — (TIF) [file pone.0129274.s017.tif]

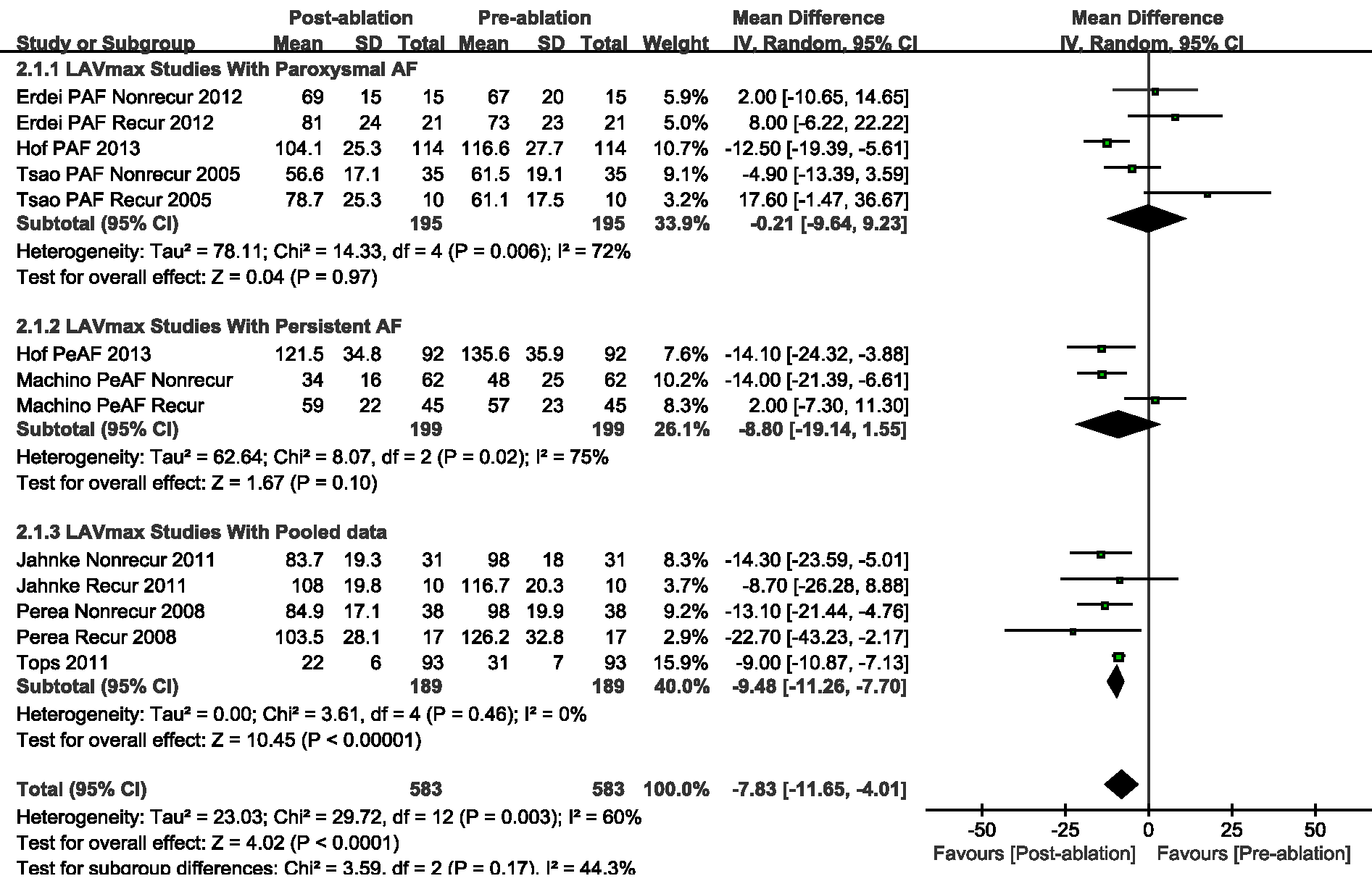

Supplement: S17 Fig — (TIF) [file pone.0129274.s018.tif]

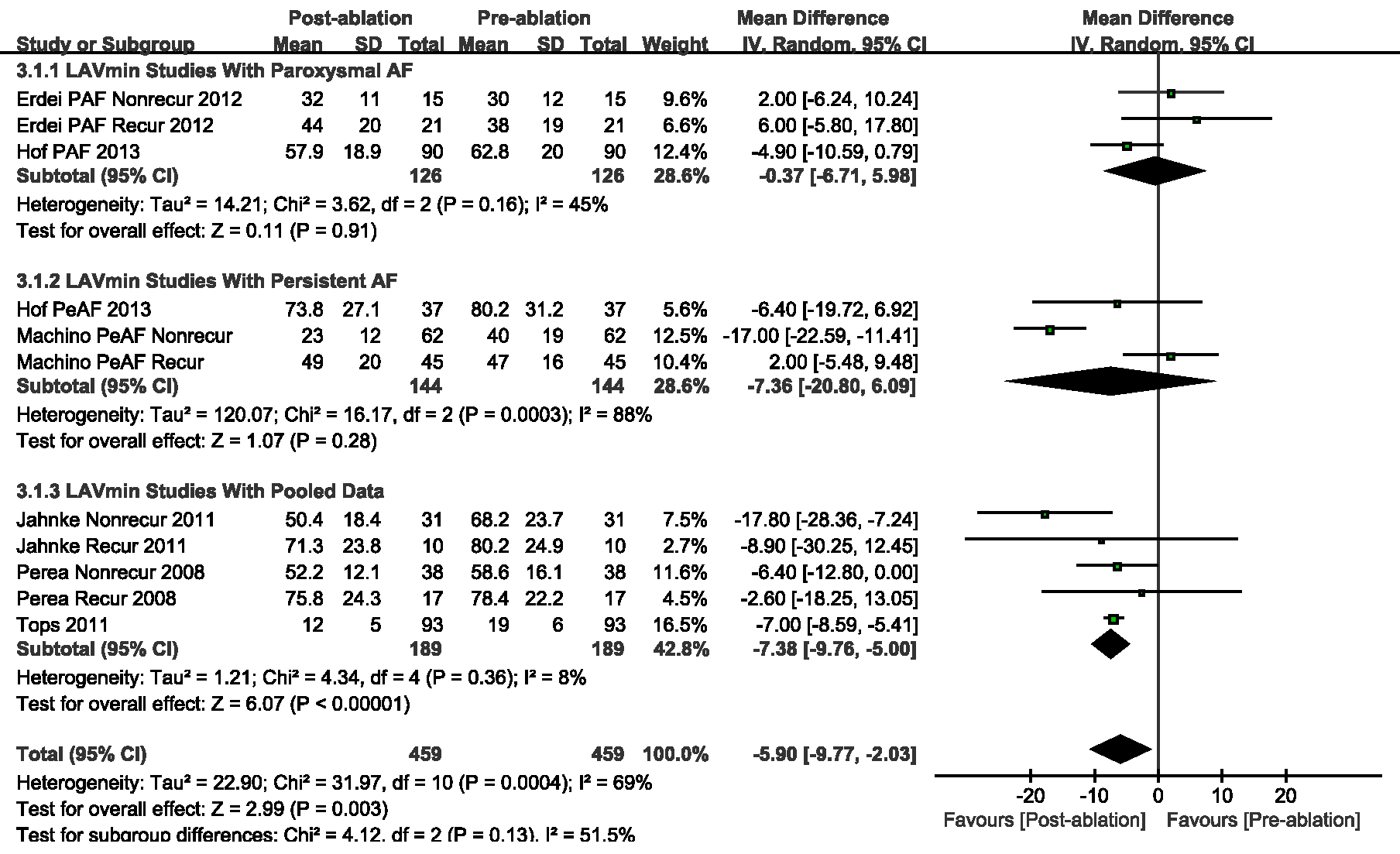

Supplement: S18 Fig — (TIF) [file pone.0129274.s019.tif]

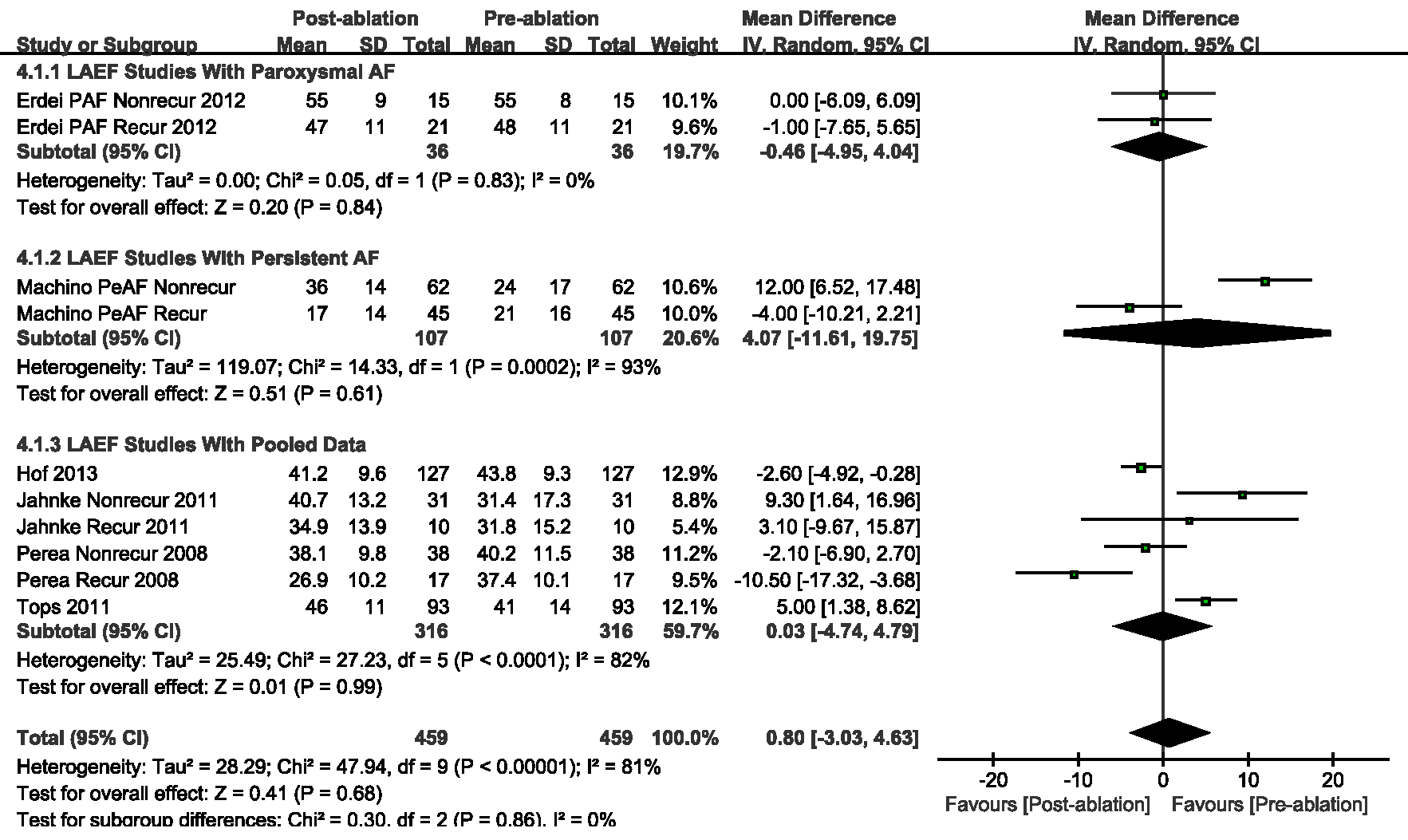

Supplement: S19 Fig — (TIF) [file pone.0129274.s020.tif]
